# Hierarchical porous photosensitizers with efficient photooxidation

**DOI:** 10.1038/s41467-023-38283-1

**Published:** 2023-05-02

**Authors:** Yajun Fang, Yuntian Yang, Rui Xu, Mingyun Liang, Qi Mou, Shuixia Chen, Jehan Kim, Long Yi Jin, Myongsoo Lee, Zhegang Huang

**Affiliations:** 1 PCFM and LIFM Lab, School of Chemistry, Sun Yat-sen University, Guangzhou, 510275 P.R. China; 2 Department of Chemistry, National Demonstration Centre for Experimental Chemistry Education, Yanbian University, Yanji, 133002 P.R. China; 3 Pohang Accelerator Laboratory, Postech, Pohang, Gyeongbuk Korea; 4 Department of Chemistry, Fudan University, Shanghai, 200438 P.R. China

**Keywords:** Self-assembly, Supramolecular polymers, Organic molecules in materials science

## Abstract

Photosensitizers (PSs) with nano- or micro-sized pore provide a great promise in the conversion of light energy into chemical fuel due to the excellent promotion for transporting singlet oxygen (^1^O_2_) into active sites. Despite such hollow PSs can be achieved by introducing molecular-level PSs into porous skeleton, however, the catalytic efficiency is far away from imagination because of the problems with pore deformation and blocking. Here, very ordered porous PSs with excellent ^1^O_2_ generation are presented from cross-linking of hierarchical porous laminates originated by co-assembly of hydrogen donative PSs and functionalized acceptor. The catalytic performance strongly depends on the preformed porous architectures, which is regulated by special recognition of hydrogen binding. As the increasing of hydrogen acceptor quantities, 2D-organized PSs laminates gradually transform into uniformly perforated porous layers with highly dispersed molecular PSs. The premature termination by porous assembly endows superior activity as well as specific selectivity for the photo-oxidative degradation, which contributes to efficient purification in aryl-bromination without any postprocessing.

## Abstract (web summary)

Photosensitizers (PS) with nano- or micro-sized pore provide a great promise in the conversion of light energy into chemical fuel but pore deformation and blocking often decreases the catalytic efficiency. Here, the authors prepare an ordered porous PSs by cross-linking hierarchical porous assemblies of hydrogen donating PSs and functionalized acceptors.

## Introduction

Developing highly efficient and stable PSs holds great promise for converting the radiant energy of sunlight into physiological chemical energy in the range of chemical oxidation to photodynamic therapy^1–3^. In photo-oxidation, the PSs can help molecular oxygen convert into singlet oxygen (^1^O_2_) or highly reactive oxygen species (ROS) by harvesting light energy^4,5^. Among them, the organic PSs have been rapidly developed due to their high extinction coefficients (*ε*), easy handling, tunable energy levels via molecular structures, and available resources^6–9^. To enhance the photosensitizer’s performance, several strategies, such as attaching heavy atoms, reducing singlet-triplet energy gap, and attenuating non-radiative decay, have been successfully employed in the catalytic processes^10–12^. Even though it is unavoidable for the reaction between the oxidative species and photocatalysts to induce several deficiencies, including lack of long-term stability, deactivation, or constant leaching, as well as low recyclability, which limit their capacity and applications^13–15^.

Spontaneous pumping of active species from the catalytic center into the reaction target is the key to extending photocatalytic stability with the efficient generation of ROS^16,17^. In biological systems, porous proteins with gating motion lead to various vital processes for the efficient capture, transport, and release of active species^18^. A typical example is provided by the uniporters that only catalyze the translocating substrate through concentration gradient^19^. The translocating process for a uniporter to complete one catalytic cycle is regulated by open-closed gating of pores at the top and bottom ends of symmetric proteasome^20^. Inspired by nature, artificial pores with the decoration of preformed PSs onto the surface of porous materials or into porous backbone have been designed as advanced materials for photo-oxidation since they can provide large accessible surface area allowing the rapid transport of ^1^O_2_ from generating sites^21,22^. However, the PSs attached to the porous surfaces have high surface energy and tend to aggregate into large particles, leading to a gradual decrease of the catalytic activity during continuous reactions^23^. While in the latter case, the shape and size are hard to control, and the embedding of PSs in the pores would further block ^1^O_2_ permeation, giving rise to unreliable catalytic activities^24^. Hydrogen-bonded macrocycles, as a new class of developed porous structures, are ideal candidates for the construction of porous materials with uniform pore size by the lateral cross linking^25,26^. Particularly, the hexameric rigid macrocycles based on the self-association of C_3_-symmetric *N*,*N’*,*N”*-tris(3-methylpyridyl) trimesic amide **1** can further aggregate into 2D honeycombed laminates through side-by-side interaction^27^. Compared with traditional rigid porous structures, the supramolecular pores based on the special hydrogen bonding possess remarkable recognition toward hydrogen donor and acceptor segments^28^. Thus, it can be anticipated that an introduction of active PSs by hydrogen bonding into macrocyclic substrates would serve as a self-assembling subunit for multi-functional porous PSs, which not only well suited for the dispersion of PS segments against the migration and aggregation but also ensure accessible pores for efficient ^1^O_2_ generation.

## Results

### Rational design and self-assembly

**Fig. 1:**
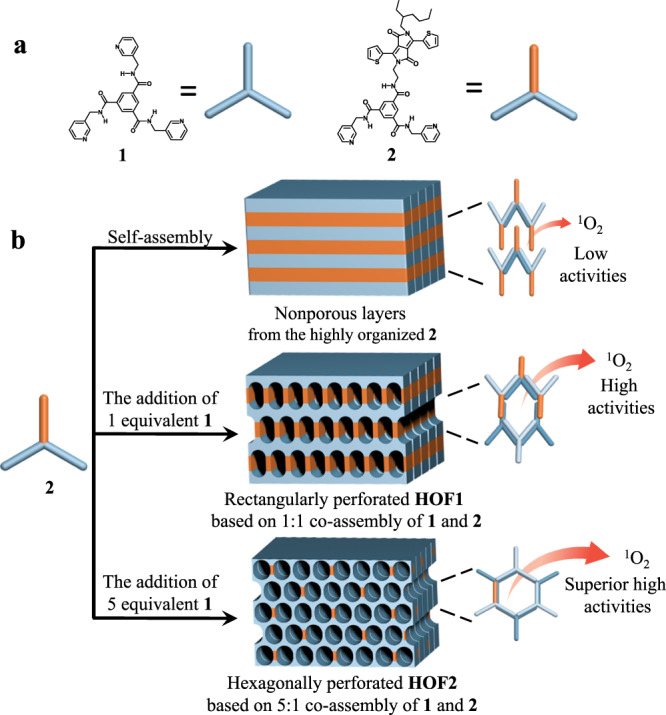
Formation and manipulation of porous photosensitizer based on hydrogen donor and acceptor. **a** Molecular structures of **1** and **2**; **b** schematic representation of the strategy for the creation of hierarchical porous photosensitizers by co-assembly of **1** and **2** (HOF hydrogen-bonded porous framework).

With this in mind, we selected thienyl-substituted diketopyrrolopyrrole (TDPP) as a model PS to substitute one edge of C_3_-symmetric **1** to obtain hydrogen donative photosensitizer **2**. The obtained molecule was characterized by ^1^H and ^13^C NMR spectroscopy and TIMS-TOF mass spectrometry, which were in full agreement with the molecular structure (Supplementary Figs. 1–3). For the existence of strong hydrogen bonding, **1** and **2** can associate hexameric macrocycles that further cross-link into homogeneously perforated laminates. Gratifyingly, the special hydrogen recognition endowed the resulting D–A co-aggregates with remarkable efficiency of ^1^O_2_ generation, which is unattainable for individual aggregation of **2** (Fig. 1).

**Fig. 2:**
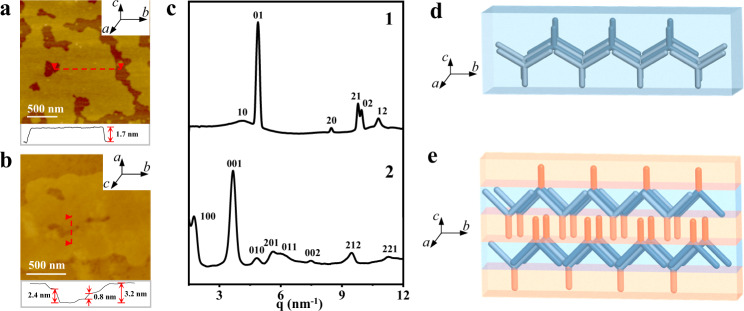
Homogeneous assembly of individual hydrogen donor and acceptor. AFM height images of **1** (**a**) and **2** (**b**). The cross-sectional profiles (bottom) were taken along the red dashed line. **c** SAXS pattern of self-assembled **1** and **2**. The samples were cast from 3 mM acetone solution after 2 days ageing. Schematic diagrams of 2D monolayer from the self-assembly of **1** (**d**) and 3D layers from the assembly of **2** (**e**).

Before the complementary complexation between **1** and **2**, the self-assembly as individual monomers was observed. Both molecules were soluble in most proton-polar solvents. However, the addition of aprotic solvents such as acetone induces the self-assembly of the nanostructures. The self-association in acetone solution was observed by vapor pressure osmometry (VPO), and Fourier transform infrared spectroscopy (FT-IR). The weights of primary aggregation based on the C_3_-symmetry **1** and **2** with the C_2_-symmetrical characteristic were measured as 938 and 1689 g mol^−1^, respectively, which were two times as large as a single molecule, suggesting that both molecules were associated with dimeric aggregates (Supplementary Fig. 4a, b). FT-IR spectra from the evaporation of 3.0 mM acetone solution showed red-shifted N–H and N = C–H stretching vibrations at 3272, 3076 cm^−1^, indicative of the existence of strong hydrogen bonding for the primary aggregates (Supplementary Fig. 4c)^29^. Atomic force microscopy (AFM) from 3.0 mM acetone solution of **1** revealed the formation of isolated sheets with the correspondent thickness of 1.7 nm, ranging in the lateral dimensions from submicrometre to several micrometers (Fig. 2a). To gain insight into the packing arrangement of molecules within the large 2D sheets, the aggregate was transferred onto thin membranes, and then the X-ray diffraction (XRD) was performed. **1** showed two kinds of periodic reflections with rectangular patterns in small- and middle-angle (Fig. 2c). Taking into account the size and dimension of dimeric aggregates, the periodicity of 1.3 nm based on (01) and (02) diffractions was approximate twice the length of dimeric wedges, suggested that neighboring molecules with the fixed amide cores zigzag hydrogen bonded each other along *b* direction to generate infinite herringbone ordering with the height of 1.7 nm. Additionally, an equal space of 1.6 nm with (12) and (21) reflections was observed in the middle angle, indicating that these herringbone alignments were packed into a fully interdigitated monolayer laminate (Fig. 2d)^30^.

In great contrast, molecule **2**, with a decoration of a TDPP segment at one apex, displayed a rather strong self-assembly tendency for the formation of thick sheets with a thickness of 3.2 nm, which was reflected in AFM measurements (Fig. 2b). Albeit rare, a few cracks with an equal thickness of 0.8 nm could be observed in the images, revealing that the thick sheets originated from bilayers stacking. The small-angle X-ray diffraction of **2** showed three strong reflections together with a number of low intensity at higher angles, indicative of the existence of a highly ordered nanoscopic structure with three distinct lattice parameters (Fig. 2c). These reflections can be indexed as a 3D primitive orthorhombic structure with lattice parameters *a* = 3.2 nm, *b* = 1.3 nm and *c* = 1.7 nm. Among them, the equidistance of 3.2 nm from small angle diffraction corresponds to inter-plane ordering, which is well-matched with the thickness of flat sheets from AFM experiments. Meanwhile, the remained lattices in small- and middle angles can be assigned as rectangular plates with corresponding constants of 1.3 and 1.7 nm. Considering the observed layer thickness of about 0.8 nm, it could be easily estimated that one unit cell consisted of two molecules^31^. These results indicated that the photosensitizer **2** again formed the herringbone super cores through extended hydrogen bonding, which was further oriented horizontally along *a* direction to generate a thin laminate with a zigzag configuration, similar to the self-organized layers from the assembly of bent-shaped aromatic amphiphilic molecules^32^. For the regular alignments of the TDPP segments with 1.3 nm distance on both the upper and downsides, the laminate had a high degree of crystallinity by interlocking, resulting in a hierarchically assembled 3D structure through strong π-π interaction (Fig. 2e).

### Co-assembled porous photosensitizers by hydrogen recognition

**Fig. 3:**
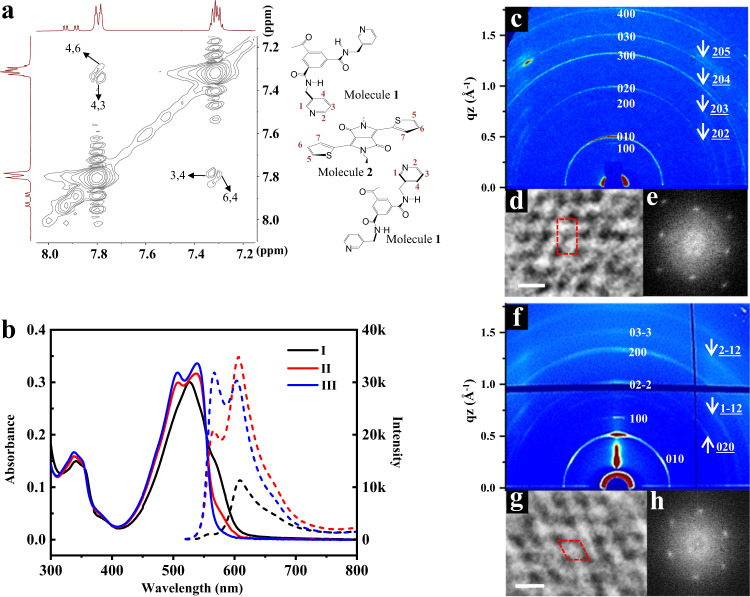
Hydrogen recognition and porous architectures by co-assembly. **a** 2D NOESY spectrum of **2** in acetone-d_6_ (3 mM) with 1 equivalent **1**. **b** UV–vis and FL spectra of **2** in acetone solution (3 mM) with and without the addition of **1**. Individual **2** (I), with the addition of 1 equivalent **1** (II) and the addition of 5 equivalent **1** (III). 2D XRD pattern (**c**), HR-TEM (**d**) and corresponding electron diffraction (**e**) of **2** from acetone solution (3 mM) with 1 equivalent **1**. 2D XRD pattern (**f**), HR-TEM (**g**) and corresponding electron diffraction (**h**) of **2** from acetone solution (3 mM) with 5 equivalent **1**. The scale bar in (**d**) and (**g**) is 2 nm.

The synergistic non-covalent interactions for creating hierarchical assembly suggested that the monolayer laminate of **1** with the pyridine exterior would intercalate into the amide functionalized aromatic core through strong hydrogen bonds, inducing a special reorganization of aromatic segments to create unique nanostructures (Supplementary Fig. 5)^33^. On this basis, the co-assembly of **1** and **2** was conducted. Upon mixing acetone solution of **1** and **2** equally, proton nuclear magnetic resonance (^1^H-NMR) showed hydrogen bonded N-H resonances at 8.35 and 8.73 ppm, indicative of the coexistence of self-association of individual **1** and **2** (Supplementary Fig. 6a). Nevertheless, the protons in the pyridine segment of **1** were considerably shifted downfield followed with the deshielding of dissociative N-H resonance of **2**, suggesting the formation of strong hydrogen bonding between both aggregates (Supplementary Fig. 6b)^34^. Indeed, the coexistence induced a strong NOE correlation between aromatic thiophene and pyridine segments, demonstrating that **1** as guest objects are intercalated within chromophore segments through strong hydrogen bonding (Fig. 3a and Supplementary Fig. 7).

**Fig. 4:**
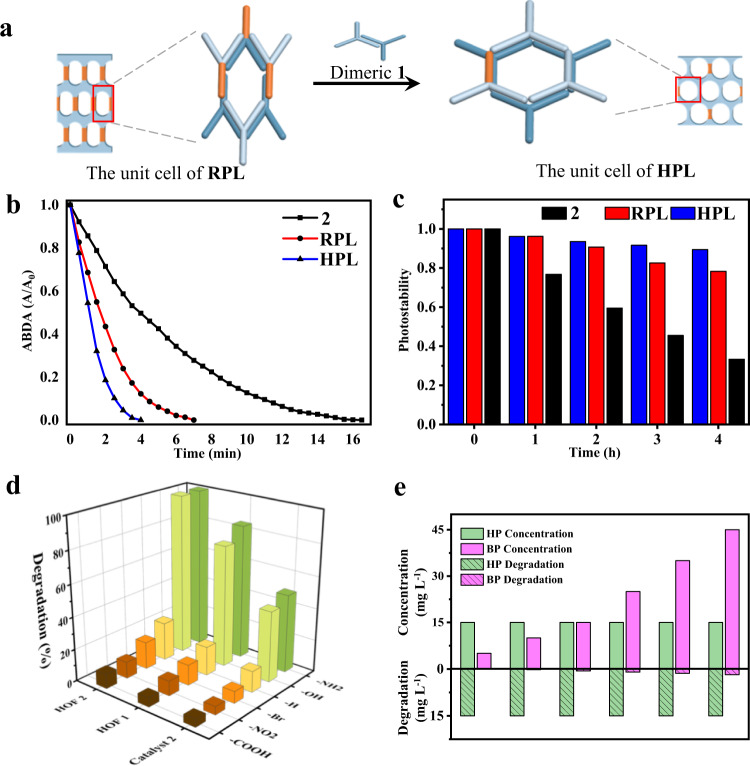
Hierachical porous frameworks and photocatalytic performance. **a** The proposed illustration for the transformation between porous structures. **b** The detection of ^1^O_2_ generation by the degrading of ABDA indicator in the presence of **2,** **RPL** and **HPL**. **c** The trace of ^1^O_2_ generation efficiency of **2,** **RPL** and **HPL** within 4 h under AM 1.5 G irradiation. **d** The degradation of pyrene derivatives by **2,** **HOF1** and **HOF2** after 50 min irradiation. **e** The degrading comparison of HP and BP in the presence of **HOF2** by the increasing BP quantity (RPL rectangularly perforated layers, HPL hexagonally perforated layers, HOF hydrogen-bonded porous framework).

The intercalation enforced a slipping of the chromophoric segment into a looser packing arrangement to minimize structural crowding at the inner of concave strips by external addition^35^. This mechanical sliding was visualized by UV-vis and fluorescence experiments (Fig. 3b). Upon titration of solution **1**, the absorption maximum of **2** centered at 525 nm was red-shifted, and the fluorescence intensity apparently increased up to 50 mol% addition of **1**, implying that the fully overlapped H-type solid changed into a looser J-type aggregate (Supplementary Fig. 8a, b)^36^. To identify the changed aggregates by co-assembly, TEM and two-dimensional X-ray diffraction were further performed with a thin membrane. The 2D laminates still maintained structural integrity even after the addition of guest **1**, which was reflected in the periodicity of rectangular characteristics with the correspondent lattices of 1.6 and 1.3 nm in the meridian (Fig. 3c). However, the diffraction contributed inter-plane ordering from the thick membrane moved to small angle with an increased *c* lattice of 2.7 nm, in contrast to pure **1** before guest addition. When the samples were cast onto the carbon-coated copper grid and then negatively stained with uranyl acetate, the TEM image showed in-plane ordered pores with a base-centered rectangular symmetry in which dark nanosized stains were regularly arrayed in light domains (Fig. 3d and Supplementary Fig. 9a)^37^. The interdomain distances were measured to be approximately 1.2 and 2.7 nm, which were consistent with the parameters obtained from X-ray scattering along *b* and *c* directions (Fig. 3e). These results demonstrate that external addition of **1** by strong hydrogen bonding triggers photosensitive solid crystals inflate into rectangularly perforated layers (RPL) by the sliding of PSs segments through the creation of internal cavity. Notably, the addition of excess guests up to 5 equivalent readily solubilize J-stacked chromophores with an optimization of porous structures (Supplementary Fig. 9b). X-ray scattering from co-assemblies with 83 mol% of **1** revealed a 2D hexagonally perforated layers (HPL) with pore distance of 1.5 nm (Fig. 3f–h). Interestingly, the co-assemblies with 50–83 mol% of **1** showed a gradual shift of the emission toward low wavelength, eventually resulting in the molecular emission of **2** (Supplementary Fig. 8d). The premature termination of PSs aggregation with porous architecture may be very beneficial for ^1^O_2_ generation^38^. The ^1^O_2_ generation efficiency was monitored by the reduced absorbance of 9,10-anthracenediyl-bis(methylene)dimalonic acid (ABDA), a commercially available ^1^O_2_ indicator. As shown in Fig. 4b, it took 16.5 min to degrade 100 nmol ABDA by 0.1 μmol **2** under the irradiation of a xenon lamp (AM 1.5 G, 100 mW cm^−2^). Meanwhile, the consumption of ABDA only needed 7 min and 4 min for RPL and HPL, respectively, indicating that the rapid generation of ^1^O_2_ was attributable to its special hollow topology based on the effectively prevented aggregation of the photosensitizer chromophores. The advanced performance was also reflected in the photo stability. In contrast to significantly quenched **2** after AM 1.5 G irradiation for 4 h, the ^1^O_2_ generation efficiencies of RPL and HPL were nearly 80% and 90% of their original values, respectively, indicating the excellent photo-stability of porous frameworks in terms of ^1^O_2_ generation was attributable to the permeability of the porous structure to gas species (Fig. 4c). To illustrate the evolution of porous structures, VPO experiments with both porous layers were further conducted. The molecular weight of RPL based on the 1:1 co-assembly was measured to be 4064 Da, denoting that the porous unit was constructed by the association of trimeric **1** and **2** (Supplementary Fig. 10a). Whereas HPL originated from 1:5 co-assembly gave 3220 Da (Supplementary Fig. 10b). The difference was close to two times of mass subtraction between **1** and **2**, indicating that the unit pore of RPL was substituted by dimeric hydrogen acceptor with the addition of excess **1** (Fig. 4a). The formation of porous layers is also illustrated by molecular dynamics simulation of 6 molecules hydrogen bonded each other along rigid wedges. Based on the composition of the porous unit from molecular weight, the unit pore of RPL was constructed by trimeric **1** and **2**, whereas pentameric **1** and individual **2** gave HPL units. The optimization shows two kinds of cyclic clusters with bi-layers after the formation of full hydrogen bonding of six molecules (Supplementary Fig. 10c, d). The cycle-like structure is energetically favorable for HPL pore, and the pore distance was calculated as 1.4 nm. On the other, the unit pore of RPL shows an ellipsoid cycle with the correspondent lattice 1.3 and 1.8 nm, which is consistent with the periodicity from the X-ray scattering. The unique regulation of the porous surrounding can be explained by the considerable exchange of hydrogen donors or acceptors towards the formation of strong hydrogen bonding. This special hydrogen bonding ability was quantified by determining the association constants (*K*_a_) of individuals **1** and **2** in acetone solution^39^. By increasing concentration up to 3.0 mM, both molecules showed a largely downfield shift in the NMR measurements due to the formation of hydrogen-bonded aggregates, respectively (Supplementary Fig. 11a, b). NMR titrations within these concentrations provided the corresponding *K*_a_ of 3264 M^−1^ for **1** and a smaller value of 2098 M^−1^ for **2** (Supplementary Fig. 11c, d).

### Cross-linked porous frameworks with excellent photosensitization effect

Due to the existence of very ordered pyridines on the porous surface, these perforated laminates could easily cross-link with 1,4-bis(bromomethyl)benzene (BBMB), resulting in the hydrogen-bonded porous frameworks (HOFs) (Supplementary Fig. 12)^40^. As shown in Supplementary Figure 13, there exists a periodical diffraction with 2*θ* ≈ 6.8° in the experimental PXRD pattern of the **HOF1** and **HOF2** based on the polymerization of 1:1 and 1:5 co-assembles. Since the crosslinking occurs on the interface of channels, the perforated structure was still retained during the polymerization, which was reflected in the peak indexing and the pore distance (Supplementary Fig. 13c). Similar to many hydrogen-bonded porous frameworks reported previously, the HOFs difficultly activated in nitrogen sorption due to the dynamic nature of hydrogen-bonding by thermo-triggers (Supplementary Fig. 14b, c), which was confirmed from differential scanning calorimetry (DSC) heating and cooling scans^41^. The traces from the first heating of both HOFs showed enthalpy changes at 81 °C and 127 °C, respectively. However, the second heating of **HOF1** after one heating and cooling scan gave an additional trace at 102 °C, while **HOF2** showed additional depression at the transition of 75 °C. These differences from first and second heating reviewed the existence of phase separation between hydrogen donor and acceptor. The permeability of these two HOFs was evaluated by the adsorption of benzaldehyde from aqueous solution. Permanently porous **HOF1** and **HOF2** absorbed the benzaldehyde uptake of 151 and 435 mg g^−1^(Supplementary Fig. 14d). Based on the Langmuir adsorption isotherm, their surface areas (*S*_0_) were calculated about 122.0, 348.8 m^2^ g^−1^, respectively^42^. Owing to the high ^1^O_2_ generation and the excellent chemical stability under light irradiation, the frameworks can be used in the photocatalysis decomposition of organic waste (Supplementary Fig. 15). At first, the degradation of stable aromatic pyrene and its derivatives, such as 1-bromopyrene and 1-nitropyrene were evaluated in the suspension of porous frameworks and aggregated **2** with the same PSs contents. In contrast to individual **2,** **HOF1** and **HOF2** exhibited high efficiency for waste degradation (Supplementary Fig. 16). Compared with **2** taking 120 min to destroy 35% pyrene, **HOF1** and **HOF2** took only 70 and 50 min, respectively, indicating that the enhanced degradation of both frameworks was attributable to the excellent permeation for active ^1^O_2_ into reactive sites. This excellent degradation was further examined with much more stable pyrene bromide and pyrene nitrite, which were stabilized even in the presence of **2** under light irradiation. However, under 1 h light irradiation, 15% or 17% of 1-boromopyrene was decomposed by the treatment of **HOF1** or **HOF2**. In spite of well-ordered porous structures for ^1^O_2_ permeation, both porous frameworks exhibited a distinct difference in the rapid photo-degradation for electron-rich aromatic wastes. Compared with **HOF1** taking 80 min for amino pyrene and 90 min for hydroxy pyrene to reach complete destruction, **HOF2** took only 40 and 50 min, respectively, for the high ^1^O_2_ generation by the optimized catalytic structure based on the efficient prevention of PSs aggregates within the porous surface.

The marked distinction in degradation for aromatic compounds motivated us to explore the possibility of selective photocatalysis over the porous frameworks (Fig. 4d)^43^. Accordingly, the photocatalytic degradations of 1-hydoxypyrene (HP) with different concentrations of 1-bromopyrene (BP) have been carried out (Fig. 4e and Supplementary Fig. 17). As shown in Fig. 4e, the targeted aromatic pyrenol was rapidly degraded with **HOF2** under the light irradiation, while the catalytic activity of **HOF2** for decomposing BP was very low. In the mixture of 15 mg L^−1^ HP and 5 mg L^−1^ BP, the photocatalytic degradation ratio of HP reached 99%, whereas only about 2% of BP was degraded under light irradiation for 30 min. Although the degradation ratio of BP had been increased to 5% within 30 min by the increment of BP concentration to 45 mg L^−1^, the decomposition of HP also retained up to 99%. These results reveal that **HOF2** can recognize the targeted pollutants from the mixture efficiently regardless of whether with or without a higher concentration than others. Hence this selective recognition was deeply used in the purification of bromo-substitution. It is known that the substitution of pyrenol by phosphorus tribromide gave a 60% conversion due to low reaction activity. After completing the reaction, the solution was readily treated with **2,** **HOF1** and **HOF2** without additional purification. Under light irradiation, the product BP can be degraded by about 39% and 20% by **2** and **HOF1**, respectively, as the complete destruction of reagent HP (Supplementary Fig. 18). Remarkably, when the irradiation was carried out by using **HOF2**, the product of BP was obtained up to 96% without any HP residue.

## Discussion

As a result, a new strategy to form hierarchically ordered porous photosensitizers has been proposed by the coassembly of hydrogen acceptor **1** and hydrogen donor photosensitizer **2**. In contrast to 2D or 3D heterogeneous porous photosensitizers relying on a series of interactions, the special recognition of hydrogen donor and acceptor spontaneously induced uniformly perforated porous catalyzers with isolated photosensitizers, having significantly higher selectivity and superior activity for photocatalytic performance. We believe that our strategy by the integrative effect of porous structure will provide new opportunities for the rational design of photosensitizers and potential photo applications in catalysis, plasma membranes, and chemical purification.

## Methods

### TEM experiments

To investigate the porous structures by co-assembly, a drop of mixed acetone solution from **1** and **2** (1–3 mM) was placed on carbon-coated copper grids and then evaporated under ambient conditions. The samples were stained by depositing a drop of uranyl acetate onto the surface. The dried specimen was observed by using a JEM-2010HR machine operated at 200 kV. The high-resolution transmission electron microscopy experiments (HR-TEM) and selected area electron diffraction were performed by JEM-ARM200P with a 200 kV accelerating voltage. The data were analyzed using Digital Micrograph software.

### AFM experiments

Both sheet-like aggregates from the assembly of individual monomers were characterized by AFM measurement. The acetone solutions of **1** or **2** (3 mM, respectively) were prepared and then cast 20 μL on the freshly cleaved mica. After the slow evaporation under air for 6 h, the films were scanned with tapping mode. The typical settings of the AFM for the high-magnification observations were as follow: a free amplitude of the oscillation frequency of ca. 1.0–1.5 V, a set-point amplitude of 0.9–1.4 V, and a scan rate of 1.5 Hz.

### NMR experiments

In order to confirm the recognition of hydrogen donor and acceptor, the ^1^H NMR solution of **2** (3 mM in acetone-d_6_) by the variation of 0.1–9 equivalent **1** was prepared, and the chemical shifts were observed on a 400 MHz NMR spectrometer. The intercalation of aggregates **1** into self-assembled **2** was determined by the NOESY test with a 1:1 mixture of **1** and **2**. After the equivalent mixing, the correlation of aromatic thiophene from aggregate **2** transforms into thiophene to pyridine signals in the NOE experiments.

To determine hydrogen bonding ability, the association constants for **1** or **2** in acetone were calculated by non-linear curve fitting. For the fitting, a series of NMR samples of individuals **1** and **2** were prepared over a range of concentrations (0.1–3 mM). Those samples were performed on a 400 MHz NMR spectrometer, and the association constants were calculated based on the equal ${K}_{a}$ model described by Eq. (1)^44^:

1 $$\delta={\delta }_{{{{{\rm{monomer}}}}}}-\Delta \left(1+\frac{1-{\left(4{K}_{a}c+1\right)}^{\frac{1}{2}}}{2{K}_{a}c}\right)$$

where $\delta$ is the observed chemical shift, ${\delta }_{{{{{\rm{monomer}}}}}}$ is the chemical shift of monomer, ${K}_{a}$ is the association constant, $c$ is the concentration (mM) of the sample, and the $\Delta$ is the difference in chemical shift between monomer and aggregate.

### Adsorption isotherm experiments

To evaluate the maximum adsorption value, the adsorption experiments were carried out with concentration variation. Before adsorption, both HOFs were cleaned with water and soaked in liquid nitrogen for 5 min. Additionally, the samples were freeze-dried under a vacuum for 24 h. The adsorption of benzaldehyde was performed in the same procedure. A representative example is described for **HOF1** at room temperature. To 10 mL of a series of benzaldehyde aqueous solution with various concentrations ranging from 10 to 80 mg L^−1^, 500 μL suspended aqueous solution of **HOF1** (1 mg mL^−1^) loaded in the semi-permeable membrane was added and then the mixture was stirred under room temperature. After being stirred for 360 min, 0.6 mL of solution was taken out, and the residual concentration of benzaldehyde was determined by UV–vis spectroscopy under 247 nm.

The amount of benzaldehyde bound to the sorbent was determined by the following equation:

2 $${q}_{e}=\frac{({C}_{0}-{C}_{e})V}{m}$$

where *q*_*e*_ (mg g^−1^) is the amount of adsorbed benzaldehyde per gram of sorbent at the equilibrium state. *C*_*0*_ (mg L^−1^) and *C*_*e*_ (mg L^−1^) are concentrations of benzaldehyde residual in filtrates at the initial and equilibrium state, respectively. *V* (L) and *m* (g) are the volume of the benzaldehyde-containing solution and the mass of sorbent used in the study.

### Surface area calculation

The model of Langmuir monolayer liquid adsorption into effective pores was used for the theoretical calculation of monolayer adsorption capacity and give surface area^41^.

3 $${S}_{0}={\Gamma }_{{{\infty }}}\times 6.023\times {10}^{23}\times {S}_{C}$$

Where $\,{\Gamma }_{\infty }$ (mol g^−1^) is monolayer adsorption capacity, 6.023 × 10^23^ is Avogadro’s constant, and *S*_*c*_ (m^2^) is the cross-sectional area of benzaldehyde.

### Photo-induced decomposition of aromatic organic compounds

The decomposition was compared with homogeneous waste in a water and acetone mixture by the dispersion of **2,** **HOF1** and **HOF2** with the same content of the PS segment. All photo-induced catalysis was performed using the same procedures. A representative example was described for destroying pyrene in the presence of **HOF2**. The **HOF2** (0.53 mg, 0.1 μmol) was added to 1 mL pyrene solution (50 mg L^−1^, H_2_O: acetone = 1:1 v/v). The mixture was vigorously stirred under simulated AM 1.5 G irradiation (100 mW cm^−2^). The degradation process of pyrene and its derivatives were monitored by the absorbance.

## Supplementary information

Supplementary Information

## Data Availability

The authors declare that all other data supporting the findings of this study are available from the article and its Supplementary Information. All other data are available from the corresponding author upon request.
