# Supplementary material for: Hierarchical porous photosensitizers with efficient photooxidation
Source: Nat Commun. 2023 May 2;14:2503. doi: 10.1038/s41467-023-38283-1 (PMC10154327; doi:10.1038/s41467-023-38283-1)
Supplement: Supplementary file 1 — Supplementary Information [file 41467_2023_38283_MOESM1_ESM.pdf]

Hierarchical porous photosensitizers with efficient photooxidation

Yajun Fang[1, †], *Yuntian Yang*[1,2, †], *Rui Xu*[1, †], *Mingyun Liang*[1], *Qi Mou*[1], *Shuixia Chen*[1], *Jehan Kim*[3], *Long Yi Jin*[2], *Myongsoo Lee*[4] *and Zhegang Huang*[1,2]*

[1]PCFM and LIFM Lab, School of Chemistry, Sun Yat-sen University, Guangzhou 510275, P. R. China
[2]Department of Chemistry, National Demonstration Centre for Experimental Chemistry Education, Yanbian University, Yanji 133002, P. R. China
[3]Pohang Accelerator Laboratory, Postech, Pohang, Gyeongbuk, Korea
[4]Department of Chemistry, Fudan University, Shanghai 200438, P.R. China
[†]These authors contributed equally: Yajun Fang, Yuntian Yang, Rui Xu

E-mail: huangzhg3@mail.sysu.edu.cn

Supplementary Methods

Materials

Trimesic acid, 3-aminopyridine, ethyldiisopropylamine, 2-(1H-Benzotriazole-1-yl)-1,1,3,3-tetramethyluroniumhexafluorophosphate (HBTU), 2-ethylhexyl bromide, 2-(Boc-amino)ethylbromide, potassium *tert*-butoxide, trifluoroacetic acid, super dried *N,N'*-dimethylformamide (DMF) from Energy Chemical, 3,6-Di(2-thienyl)-2,5-dihydropyrrolo[3,4-c]pyrrole-1,4-dione from HWRK Chem were used as received. The molecule **1** was synthesized according to previous reported method[1]. Unless otherwise indicated, all reagents and materials were obtained from commercial suppliers without purification. Distilled water was polished by ion exchange and filtration.

General

Vapor pressure osmometry (VPO) experiment was recorded on OSMOMAT 07 instrument. Fourier transform infrared spectroscopy (FT-IR) experiments were carried out on the Perkin Elmer Frontier IR spectrometer using ATR mode under ambient conditions. The UV-vis spectra were obtained from a Metash UV-8000S Spectrophotometer. Fluorescence spectra were obtained from Shimadzu RF-6000 Spectrofluorophotom-eter. Dynamic light scattering (DLS) was performed by NanoBrook EliteSizer Omni of Brookhaven. ^{1}H and ^{13}C NMR spectra were recorded on Bruker Avance III 400 MHz and the Solid-state crosspolarization magic angle spinning (CP/MAS) ^{13}C NMR spectra were recorded on Bruker Avance III 400WB (400 MHz) NMR spectrometer at ambient temperature with a magic angle spinning rate of 7.0 kHz. The NOESY experiments were performed by JEOL JNM-ECZ400. The synthesized molecular weights were determined with trapped ion mobility spectroscopy-time-of-flight mass spectrometry (UltiMate3000-timsTOF). ESI-MS spectrum was recorded on a LTQ XL mass spectrometer using methanol as mobile phase. Elemental analysis was obtained from Elementar Analyzer Vario EL. X-ray photoelectron spectroscopy (XPS) measurements were performed on ESCALab250 Mark (VG) photoelectron spectrometer using a monochromatic Al Kα X-ray source. Thermal gravimetric analysis (TGA) was performed on TG209F1 under N_2 by heating to 700 °C at a rate of 10 °C min^{-1}. The AFM measurements were performed using Bruker Multimode 8 by tapping mode. X-ray scattering experiments were carried out at the 3C beamline in Pohang Accelerator Laboratory. Photocatalytic experiments were performed using PerfectLight PLS-SXE300.

Reagents and conditions: (a) potassium *tert*-butoxide, 2-ethylhexyl bromide, DMF, 6h; (b) potassium *tert*-butoxide, 2-(Boc-amino)ethylbromide, DMF, 12h; (c) trifluoroacetic acid, dichloromethane (DCM), 0°C, 3h; (d) 3-aminopyridine, ethyldiisopropylamine, HBTU, DMF, 0°C, 4h; (e) ethyldiisopropylamine, HBTU, DMF, 0°C, 8h.

Compound 5. 3,6-Di(2-thienyl)-2,5-dihydropyrrolo[3,4-c]pyrrole-1,4-dione (3.0 g, 10 mmol) in 150 mL DMF was treated with potassium *tert*-butoxide (1.68 g, 15 mmol) for 0.5 h at room temperature. Then, 2-ethylhexyl bromide (2.88 g, 15 mmol) was slowly added and the mixture was further stirred for 6h at room temperature. The solvent was removed by a rotary evaporator and the crude product was purified by column chromatography (silica gel) using DCM as eluent to yield 38% (1.57 g) of red solid.
^{1}H-NMR (400 MHz, Chloroform-D) δ 9.31 (s, 1H), 8.86 (d, *J* = 4.0 Hz, 1H), 8.37 (d, *J* = 4.0 Hz, 1H), 7.66 (d, *J* = 4.0 Hz, 1H), 7.61 (d, *J* = 4.0 Hz, 1H), 7.29 (t, *J* = 4.0 Hz, 1H), 7.23 (t, *J* = 4.0 Hz, 1H), 4.11-3.97 (m, 2H), 1.91-1.81 (m, 1H), 1.38-1.23 (m, 8H), 0.87 (m, 6H); ^{13}C-NMR (100 MHz, Chloroform-D) δ 162.38, 161.72, 140.89, 136.52, 135.39, 132.13, 130.92, 130.81, 129.92, 129.07, 128.38, 108.64, 108.43, 45.96, 39.16, 30.30, 28.41, 23.62, 23.06, 14.01, 10.53; ESI-MS: m/z calculated for $C_{22}H_{24}N_2O_2S_2$ $[M+H]^+$, 413.13; found: 413.61.

Compound 6. The potassium *tert*-butoxide (0.52 g, 4.6 mmol) was added into the DMF (75 mL) solution of compound **5** (1.57 g, 3.8 mmol) and stirred for 1 h. Then 2-(Boc-amino)ethylbromide (1.03 g, 4.6 mmol) was further added into the mixture. After 12 h vigorously stirring at room temperature, the solvent

was removed in a rotary evaporator. The crude product was purified by column chromatography (silica gel) using DCM as eluent to yield 32% (0.67 g) of red solid.
^{1}H-NMR (400 MHz, Chloroform-D) δ 8.83 (d, *J* = 4.0 Hz, 2H), 7.64 (d, *J* = 5.0 Hz, 2H), 7.28 (m, 2H), 5.03 (s, 1H), 4.23 (t, *J* = 6.0 Hz, 2H), 4.10-3.94 (m, 2H), 3.54 (t, *J* = 6.0 Hz, 2H), 1.91-1.81 (m, 1H), 1.38 (s, 9H), 1.37-1.23 (m, 8H), 0.87 (m, 6H), ^{13}C-NMR (100 MHz, Chloroform-D) δ 161.9, 135.9, 131.0, 130.9, 129.9, 129.7, 128.9, 128.6, 108.4, 107.8, 79.6, 46.1, 42.4, 40.8, 39.3, 30.4, 28.5, 28.5, 23.7, 23.2, 14.2, 10.7; UV/Vis: λ_{max} = 538 nm; ESI-MS: m/z calculated for $C_{29}H_{37}N_3O_4S_2$ $[M+Na]^+$, 578.22; found: 578.25.

Compound 7. To a solution of **6** (0.67 g, 1.2 mmol) in DCM (100 mL), 20 mL TFA was dropwided slowly at 0°C. The mixture was vigorously stirred for 3 h. After completion of the reaction, the solvent was removed in a rotary evaporator. The crude product was purified by column chromatography (aluminium oxide) using MeOH : DCM (1:10, v/v) as eluent to yield 90% (0.49 g) of red solid.
^{1}H-NMR (400 MHz, Chloroform-D) δ 8.92 (d, *J* = 4.0 Hz, 1H), 8.85 (d, *J* = 4.0 Hz, 1H), 7.64 (t, *J* = 5.0 Hz, 2H), 7.31-7.27 (m, 2H), 4.18 (t, *J* = 6.8 Hz, 2H), 4.10-3.95 (m, 2H), 3.08 (t, *J* = 6.8 Hz, 2H), 1.91-1.81 (m, 1H), 1.39-1.21 (m, 8H), 0.87 (m, 6H); ^{13}C-NMR (100 MHz, DMSO-d_6) δ 161.8, 141.0, 135.3, 130.8, 130.5, 129.8, 129.6, 128.8, 128.4, 108.3, 107.8, 46.0, 41.9, 39.1, 30.2, 28.4, 28.0, 23.6, 23.1, 14.0, 10.5, 10.7; UV/Vis: λ_{max} = 536 nm; ESI-MS: m/z calculated for $C_{24}H_{29}N_3O_2S_2$ $[M-H]^-$, 454.17; found: 454.22.

Compound 8. Trimesic acid (1.0 g, 4.8 mmol), ethyldiisopropylamine (1.04 g, 9.6 mmol) and 3-aminopyridine (1.24 g, 9.6 mmol) were dissolved in DMF (100 mL) and then cooled into 0 °C. 19 mL DMF solution of HBTU (3.64 g, 9.6 mmol) was dropwised into the reaction mixture. After stirring for 4 h, the solvent was removed in a rotary evaporator. The crude product was purified by column chromatography (silica gel) using MeOH : DCM (1:5, v/v) as eluent to yield 34% (0.64 g) of white solid.
^{1}H-NMR (400 MHz, DMSO-d_6) δ 9.32 (t, *J* = 5.8 Hz, 2H), 8.56 (s, 2H), 8.52 (s, 2H), 8.45 (d, *J* = 4.0 Hz, 2H), 8.31 (s, 1H), 7.75 (d, *J* = 8.0 Hz, 2H), 7.36 (dd, *J* = 8.0, 4.0 Hz, 2H), 4.50 (d, *J* = 5.8 Hz, 4H); ^{13}C-NMR (100 MHz, DMSO-d_6) δ 166.1, 148.9, 148.1, 135.2, 135.1, 133.9, 130.7, 127.8, 123.5, 40.6; UV/Vis: λ_{max} = 223 nm; ESI-MS: m/z calculated for $C_{21}H_{18}N_4O_4$ $[M+H]^+$, 391.13; found: 391.24.

Compound 2. Compound **8** (0.39 g, 1.0 mmol), ethyldiisopropylamine (0.13 g, 1.0 mmol) and compound **7** (0.46 g, 1.0 mmol) were dissolved in DMF (50 mL) and cooled into 0 °C. Then, 5 mL DMF solution of HBTU (0.38 g, 1.0 mmol) was dropwised into the reaction mixture and stirred for 8 h. After completion of the reaction, the solvent was removed in a rotary evaporator. The crude product was purified by column chromatography (silica gel) using MeOH : DCM (1:10, v/v) as eluent to yield 61% (0.51 g) of red solid.
^{1}H-NMR (400 MHz, DMSO-d_6) δ 9.31 (t, *J* = 6.0 Hz, 2H), 8.96 (t, *J* = 6.0 Hz, 1H), 8.71 (d, *J* = 4.0 Hz, 1H), 8.68 (d, *J* = 4.0 Hz, 1H), 8.58 (s, 2H), 8.47 (d, *J* = 5.6 Hz, 2H), 8.46(s, 1H), 8.39 (s, 2H), 8.06 (d, *J* = 4.9 Hz, 1H), 8.01 (d, *J* = 4.9 Hz, 1H), 7.74 (d, *J* = 8.0 Hz, 2H), 7.38 (dd, *J* = 8.0, 5.6 Hz, 2H), 7.33-7.31 (m, 2H), 4.51 (d, *J* = 6.0 Hz, 4H), 4.23 (t, *J* = 6.4 Hz, 2H), 3.94 (m, 2H), 3.59-3.56 (m, 2H), 1.72 (m, 1H), 1.23 (m, 8H), 0.80 (m, 6H); ^{13}C-NMR (100 MHz, DMSO-d_6) δ 166.2, 166.0, 161.3, 161.2, 149.4, 148.6, 140.1, 140.0, 135.8, 135.3, 135.3, 135.0, 135.0, 134.6, 133.1, 133.0, 129.6, 129.5, 129.3, 129.0, 129.0, 128.9, 124.0, 107.4, 107.4, 45.4, 41.8, 41.1, 39.1, 38.9, 30.0, 28.1, 23.4, 22.9, 14.3, 10.7; UV/Vis: λ_{max} = 536 nm; TIMS-TOF mass: *m/z* calculated for $C_{45}H_{45}N_7O_5S_2$ $[M+H]^+$, 828.29; found: $[M+H]^+$, 828.30.

Synthesis of hydrogen-bonded porous frameworks HOF1 and HOF2. Both frameworks were synthesized using the same procedure. A representative example is described for **HOF1**. 1,4-bis(bromomethyl)benzene (40 mg, 0.15 mmol) was added into 16 mL co-assembled acetone solution with 3.75 mM **1** and 3.75 mM **2**. After stirred for 48 h at room temperature, the obtained precipitates were collected by filtration and washed with dichloromethane and ethanol for three times. The residue was dried at 50 °C under vacuum to yield 79 mg (86 %) vermilion powders. Both HOFs were characterized

by XPS, ^{13}C NMR and elemental analysis as presented in Supplementary Figure 12 and Supplementary Table 1.
Both porous frameworks **HOF1** and **HOF2** based on the cross-linking of RPL and HPL showed remarkable chemical stability even in strong alkaline or acid solutions. After soaking in the NaOH (pH 11) and HCl (pH 3) solution, the structural periodicity of pristine HOFs were well preserved in the powder XRD measurements (Supplementary Fig. 13). Thermal gravimetric analysis (TGA) also revealed that the resulted porous frameworks were thermally stable up to 300 °C (Supplementary Fig. 14a).

Preparation of self-assembled 2, RPL and HPL.
Self-assembled catalyst from individual 2. The stock solution of **2** in acetone (3 mM) was prepared by ultrasound for 10 mins to obtain dark red solution. After a few minutes of incubation, the solution was used as self-assembled catalyst from individual **2** without further treatment.

RPL and HPL catalysts from the co-assembly of 1 and 2. Two kind of stock solutions of **1** (3 mM) and **2** (3 mM) in acetone were prepared respectively. Samples for the rectangularly perforated layers (RPL) were prepared by mixing 1 mL of both aliquots under shaking for 10 min to get an red solution. After a few minutes of incubation, the solution was used as RPL catalyst. While the samples of hexagonally perforated layers (HPL) were prepared by mixing 5 mL of aliquot **1** and 1 mL of aliquot **2** under the shaking to gain an orange solution. After a few minutes of incubation, the solution was used as HPL catalyst without further treatment.

Computation. Simulations were conducted with Materials Studio software by DFTB+ method. According to the process of co-assembly between hydrogen donor and acceptor, two kinds of hexameric macrocycles could be constructed. The connection of trimeric **1** and trimeric **2** by hydrogen bonding gives the RPL pore with rectangular symmetry. Whereas, one substitution of hexameric 1 by individual 2 result in the HPL pore. The potential energy of both cycles was then minimized until the root-mean square derivative was 1.0 (Kcal/mol)/Å or less.

The geometry optimization of RPL and HPL were performed through DFTB+ method in Materials Studio program. A self-establish model that was created to be an extension of the 3ob library of Slater-Koster was adopted for calculations. The k point gridding setting as 1x1x1. The self consistent charges (SCC) were used with SCC tolerance set to fine and eigensolver set to divide and conquer. The convergence criteria for geometry optimization and energy calculation were set to 0.05 kcal/mol for the energy tolerance,0.5 kcal/mol/Å for the maximum force tolerance and 0.01 Å for the maximum displacement tolerance. The properties basis set was used with Density of states, Electron density, Frequency, Orbitals, Population analysis.

Supplementary Figures

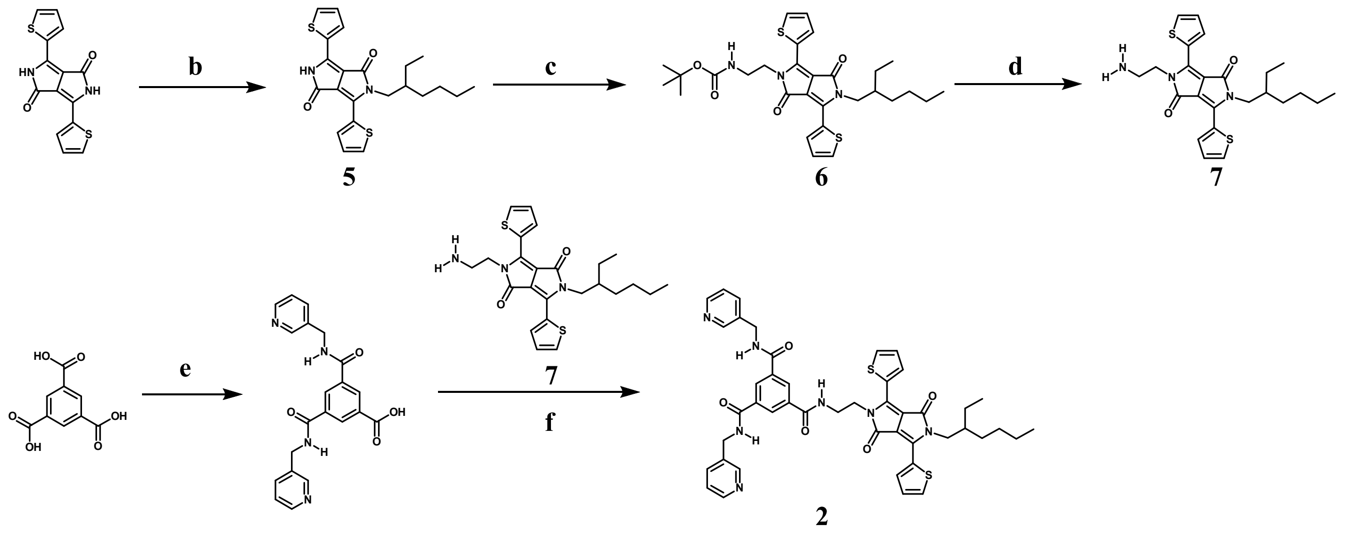

Supplementary Figure 1. Sytnesis of molecule 2.

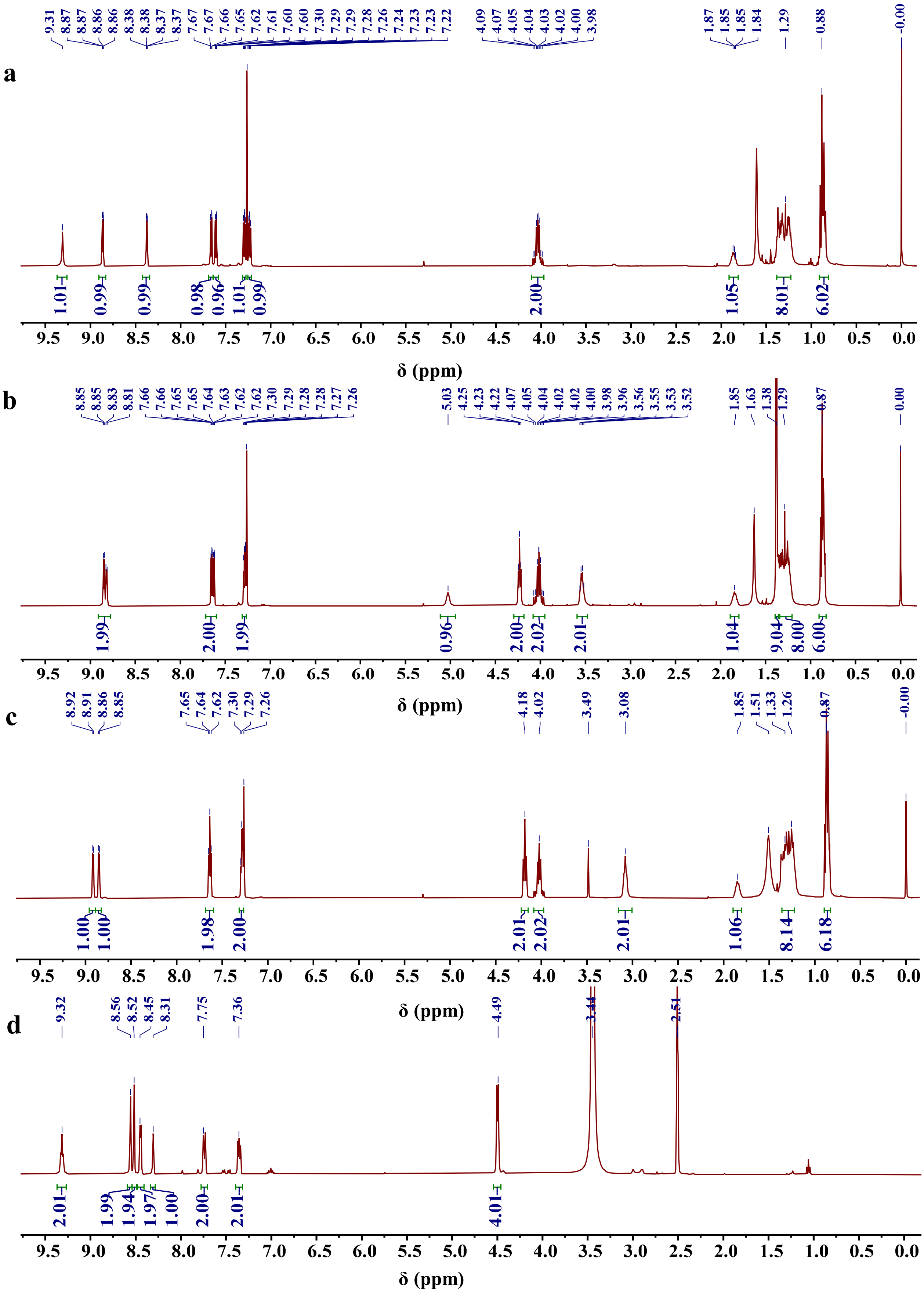

Supplementary Figure 2. ^{1}H NMR of intermediates 5 (a), 6 (b), 7 (c) and 8 (d).

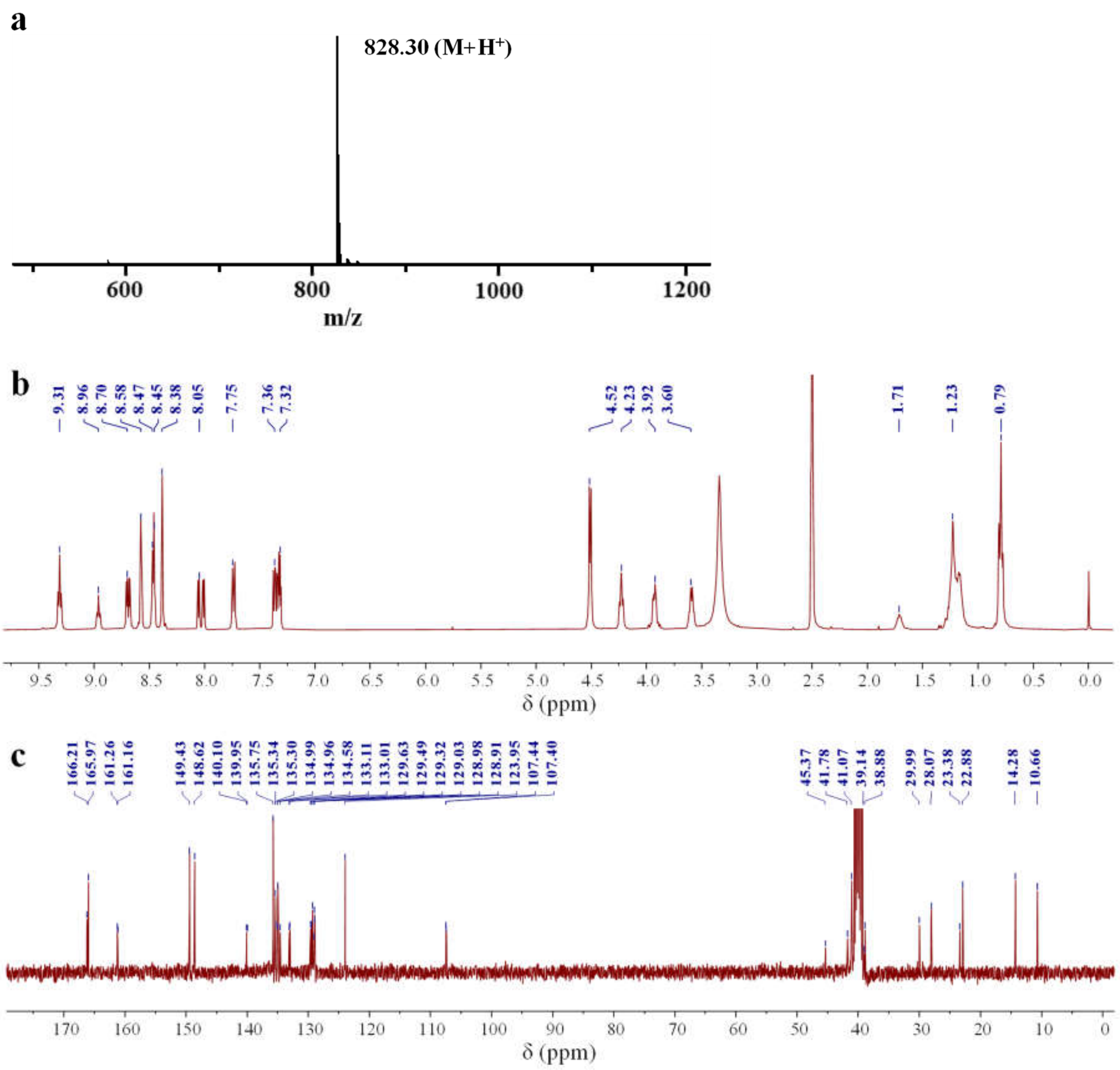

Supplementary Figure 3. (a) TIMS-TOF mass spectrum of molecule **2**. ^{1}H NMR (b) and ^{13}C NMR (c) spectra of **2** in DMSO-d_6.

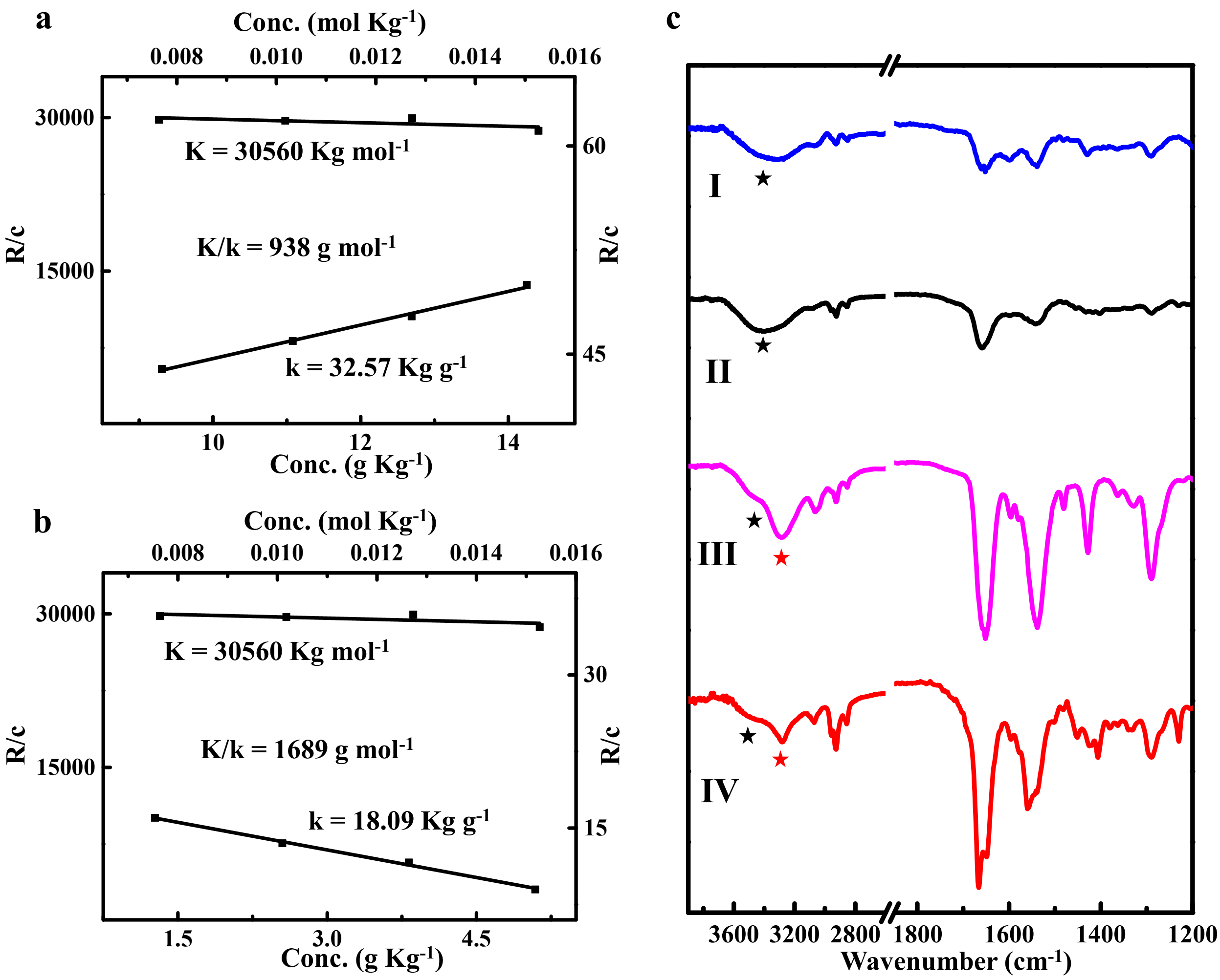

Supplementary Figure 4. VPO results of primary aggregates **1** (a) and **2** (b) in acetone using polycaprolactone (average molecular weight = 4000 g mol^{-1}) as a standard for the determination of K. The measured molecular weight of **1** as 938 g mol^{-1}, and **2** as 1689 g mol^{-1}, which were two times as large as single molecule respectively. (c) FT-IR spectra of **1** (I) and **2** (II) from the evaporation of 3 mM MeOH solution and the spectra of **1** (III) and **2** (IV) from the evaporation of 3 mM acetone solution (Black star is disassociated N-H stretching vibration, while red star is hydrogen bonded N-H stretching vibration).

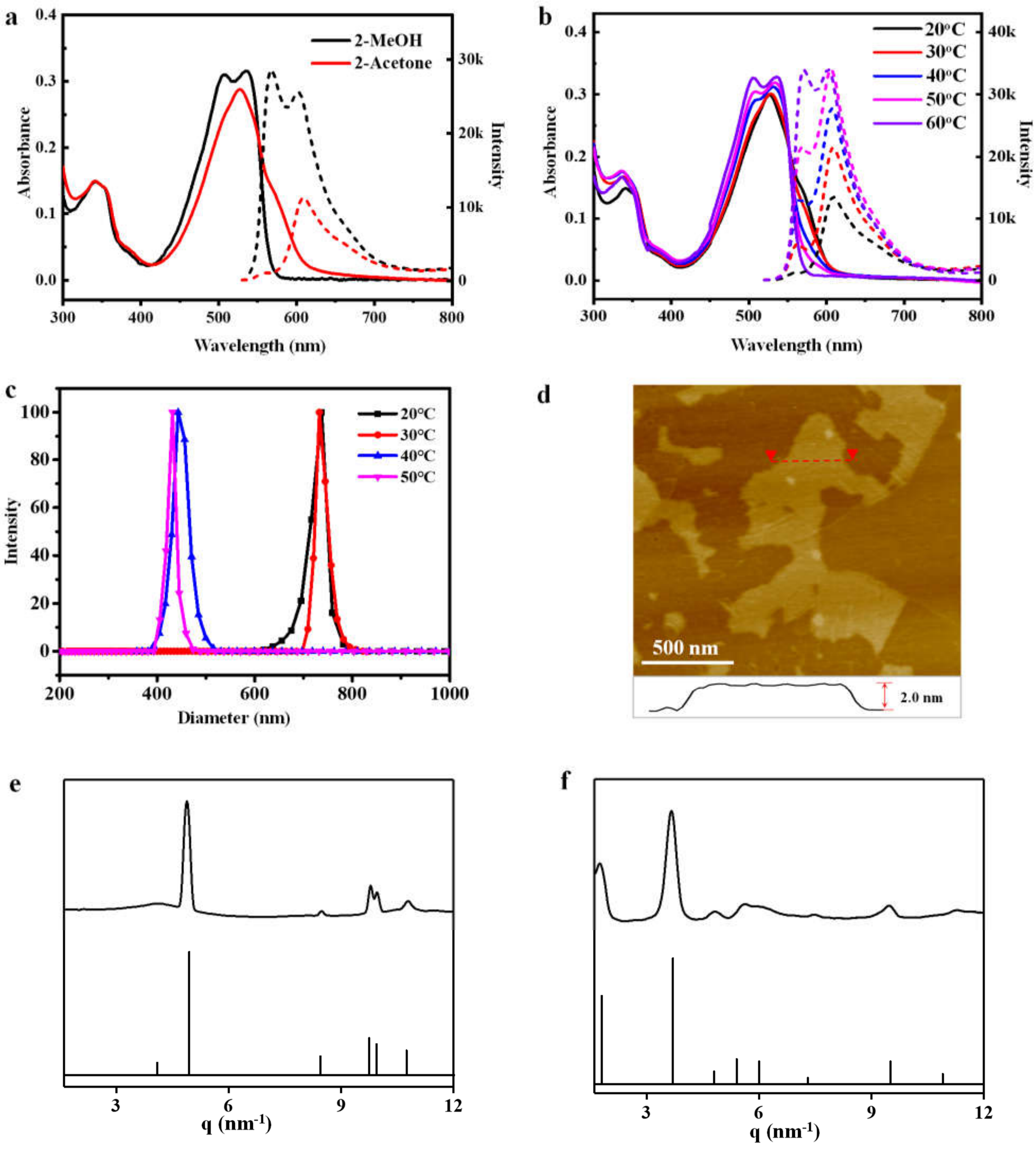

Supplementary Figure 5. (a) UV-vis (solid line) and fluorescence (dash line) spectra of **2** in MeOH (3 mM) and acetone (3 mM). (b) Temperature dependent absorption (solid line) and emission (dash line) of **2** in acetone (3 mM). (c) Size distribution graphs of **2** in acetone (3 mM) from 20 °C to 50 °C. (d) AFM height image of **2** by a slow evaporation of acetone solution (3 mM) at 50 °C. (e) X-ray diffraction and the comparison of rectangular symmetric monolayers from self-assembly of **1**. (f) X-ray diffraction and the comparison of 3D primitive orthorhombic structure from self-assembly of **2**.

The absorption maximum of **2** in acetone solution is blue-shifted and fluorescence significantly reduced with respect to those observed in methanol solution. The variation of the absorption and emission spectra were also by temperature dependent experiments in acetone solution reversibely, indicative of the photosensitizer **2** can form *H*-type aggregation in acetone solution.

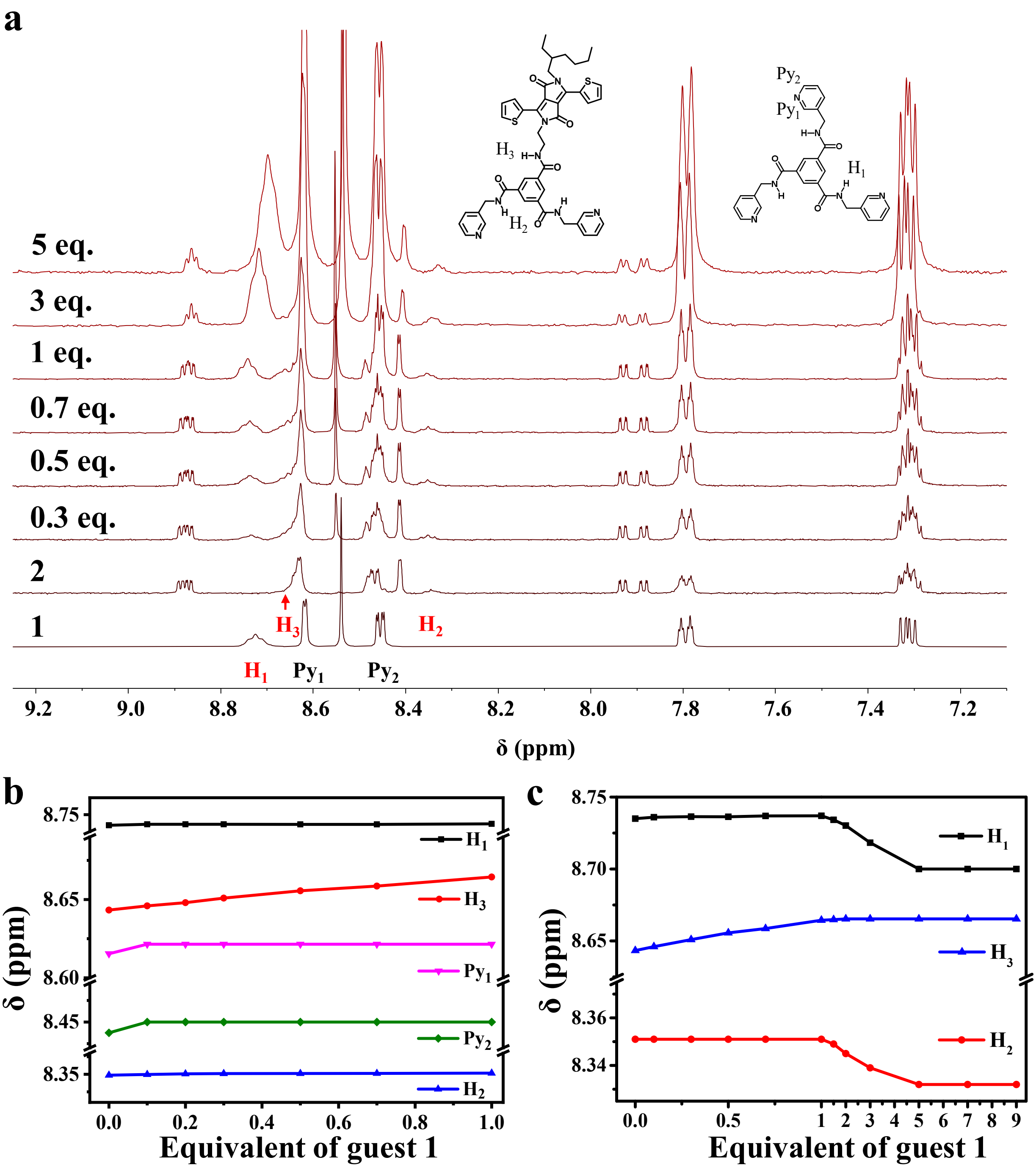

Supplementary Figure 6. (a) ^{1}H NMR spectra of **1** and **2** in acetone-d6 (3 mM) and chemical shift by the mixing through the increase of **1**. (b) The variation of chemical shifts of N-H resonances and the protons in pyridine segment in acetone-d6 by the addition of **1** up to 1 equivalent. (c) The trace of N-H resonances by the addition of excess **1**.

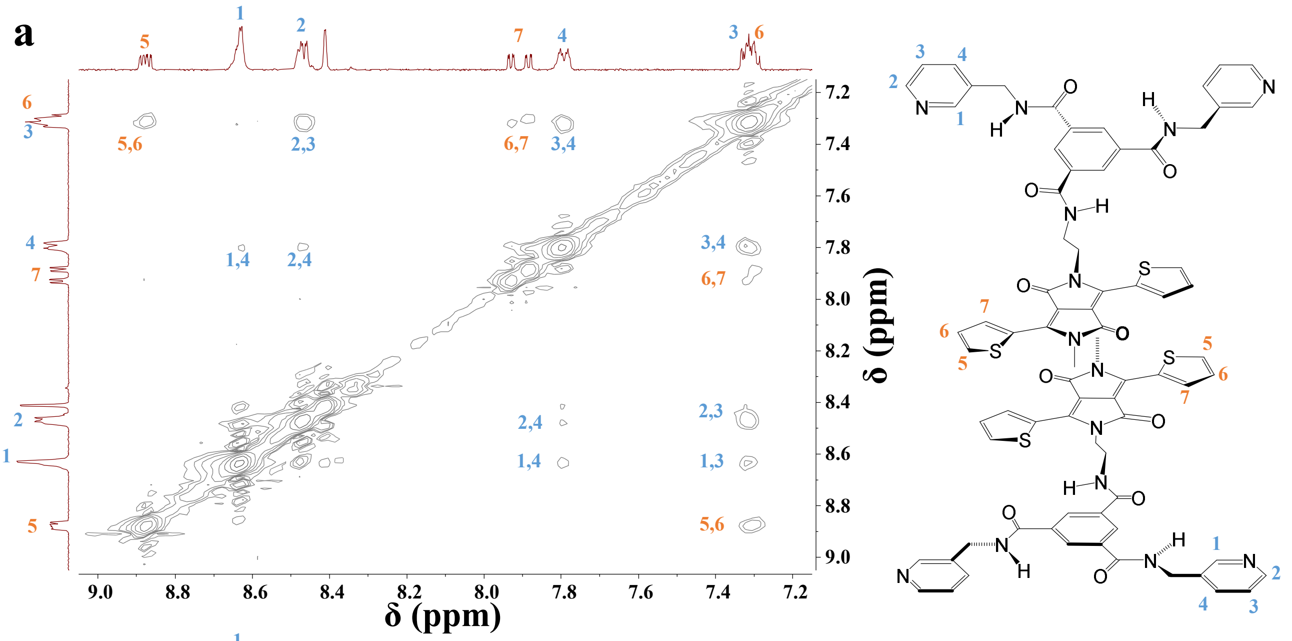

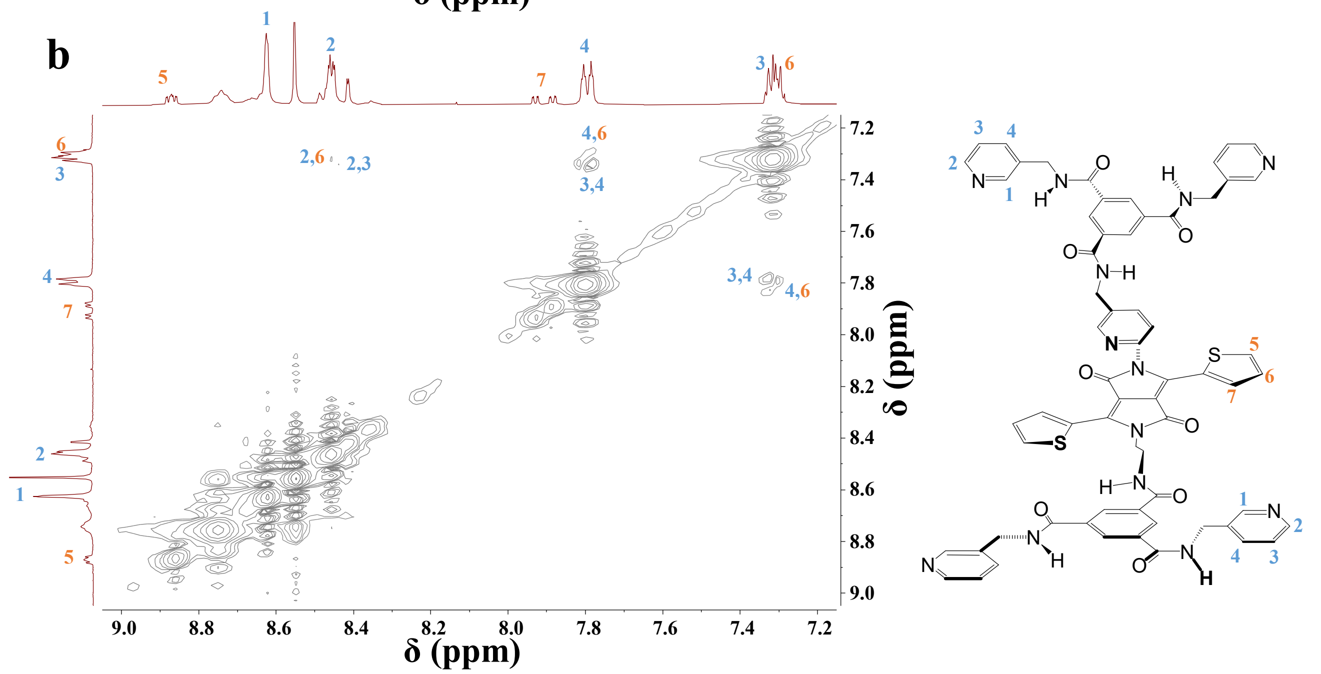

Supplementary Figure 7. 2D NOESY spectrums of **2** in acetone-d$_6$ (3 mM) (a) and the mixed solution (b) by the addition of 1 equivalent **1**.

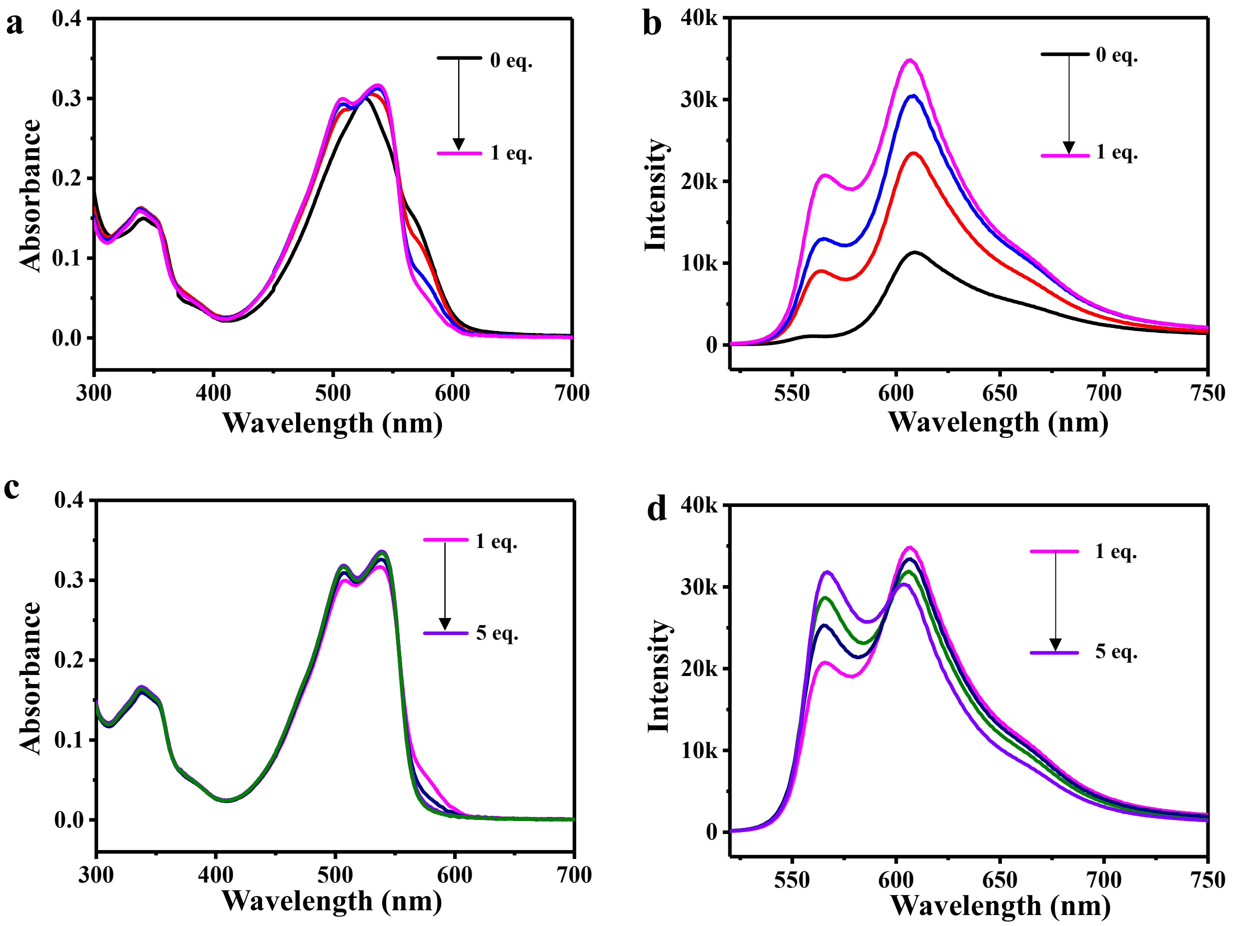

Supplementary Figure 8. The absorption (a) and emission (b) of **2** in acetone solution (3 mM) by the titration of 1 equivalent **1**. The continuous absorption (c) and emission (d) of **2** in acetone solution (3 mM) with the addition of **1** up to 5 equivalent.

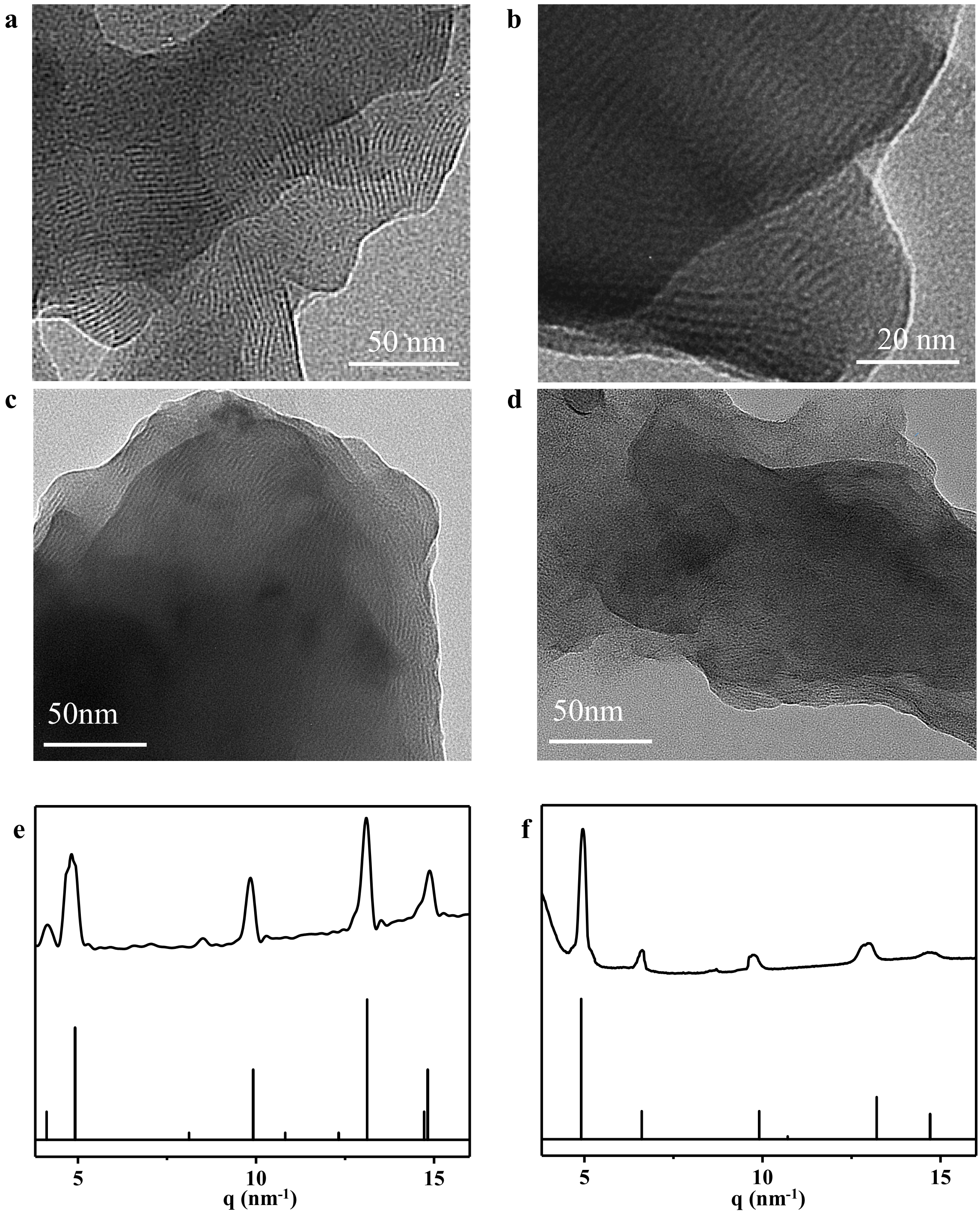

Supplementary Figure 9. TEM image of **2** from 3 mM acetone solution with the addition of 1 equivalent **1**(a) and 5 equivalent **1** (b). TEM image of **HOF1** (c) and **HOF2** (d) from aqueous dispersion (3 mM). (e) X-ray diffraction and the comparison of rectangularly perforated layers from the 1:1 co-assembly of **1** and **2**. (f) X-ray diffraction and the comparison of hexagonally perforated layers from the 5:1 co-assembly of **1** and **2** (**HOF**: hydrogen-bonded porous framework).

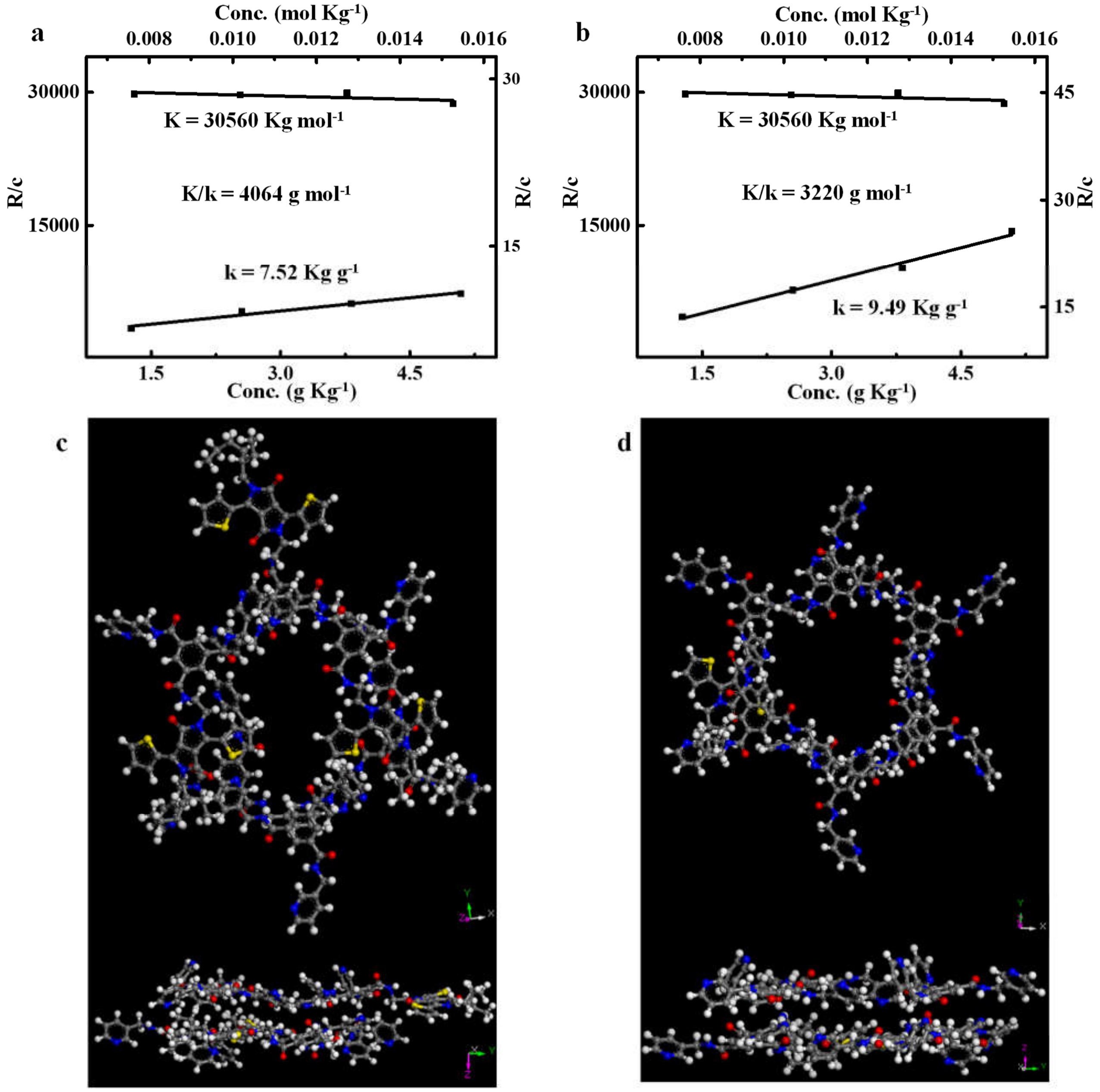

Supplementary Figure 10. VPO results of **2** in acetone solution with 1 equivalent **1** (a) and 5 equivalent **1** (b). Both measurements were used polycaprolactone (average molecular weight = 4000 g mol^{-1}) as a standard for the determination of K. The k of 1:1 co-aggregates was measured as 7.52 Kg g^{-1} and the molecular weight of RPL based on 1:1 co-assembly was calculated for 4064 g mol^{-1}. The k of 1:5 co-aggregates was measured as 9.49 Kg g^{-1} and the molecular weight of HPL based on 1:5 co-assembly was calculated for 3220 g mol^{-1}. (c) Top view (above) and side view (below) of an ellipsoid structure by simulation of RPL unit pore. (d) Top view (above) and side view (below) of a cyrcle-like structure by simulation of HPL unit pore (**RPL**: rectangularly perforated layers, **HPL**: hexagonally perforated layers, **HOF**: hydrogen-bonded porous framework).

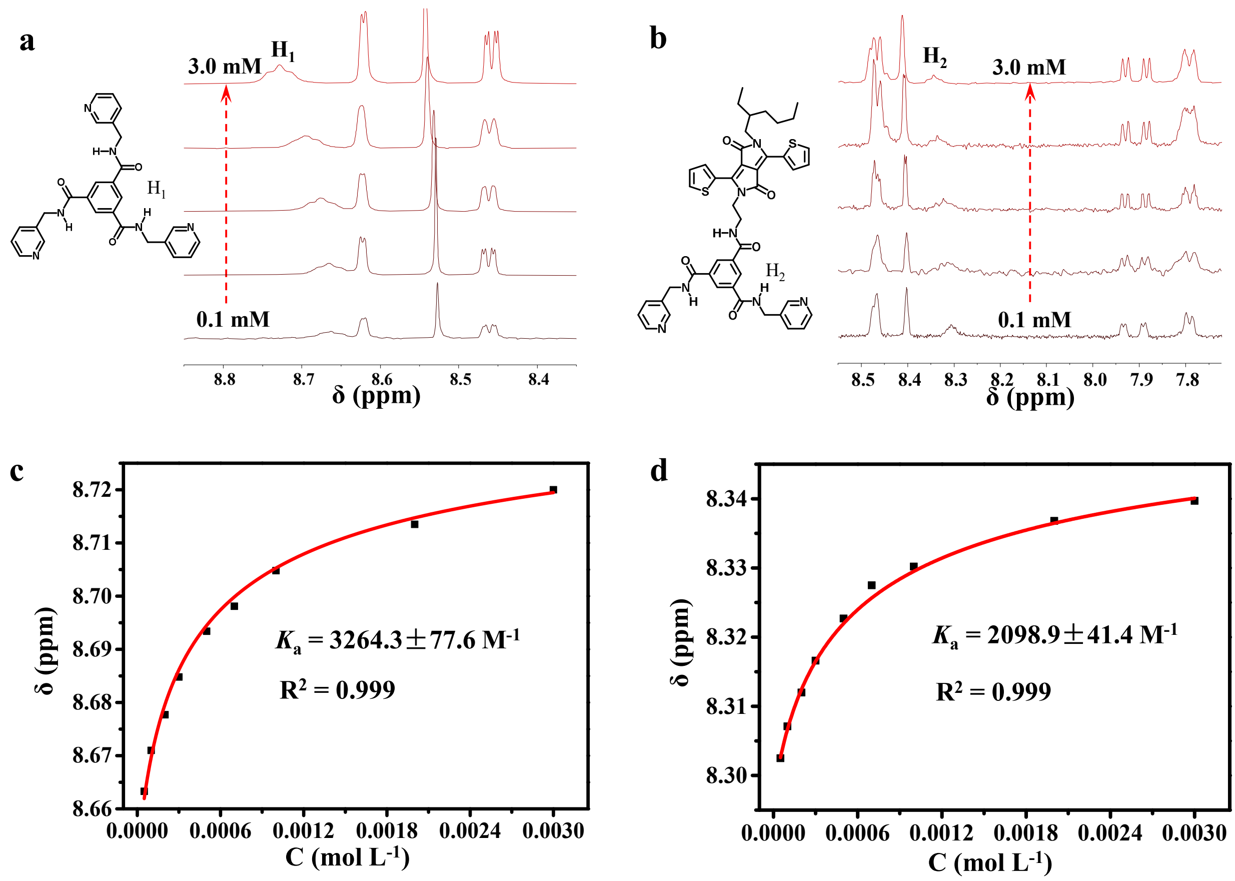

Supplementary Figure 11. NMR titrations of **1** (a) and **2** (b) in acetone-d_6 with the variation of concentration respectively. The concentration increased from 0.1 to 3.0 mM. Non-linear curve-fitting of H_1 and H_2 for the determination of the association constants (K_a) for the aggregation of **1** (c) and **2** (d) respectively. The value of K_a was calculated by the equation (1) and shown in the figures.

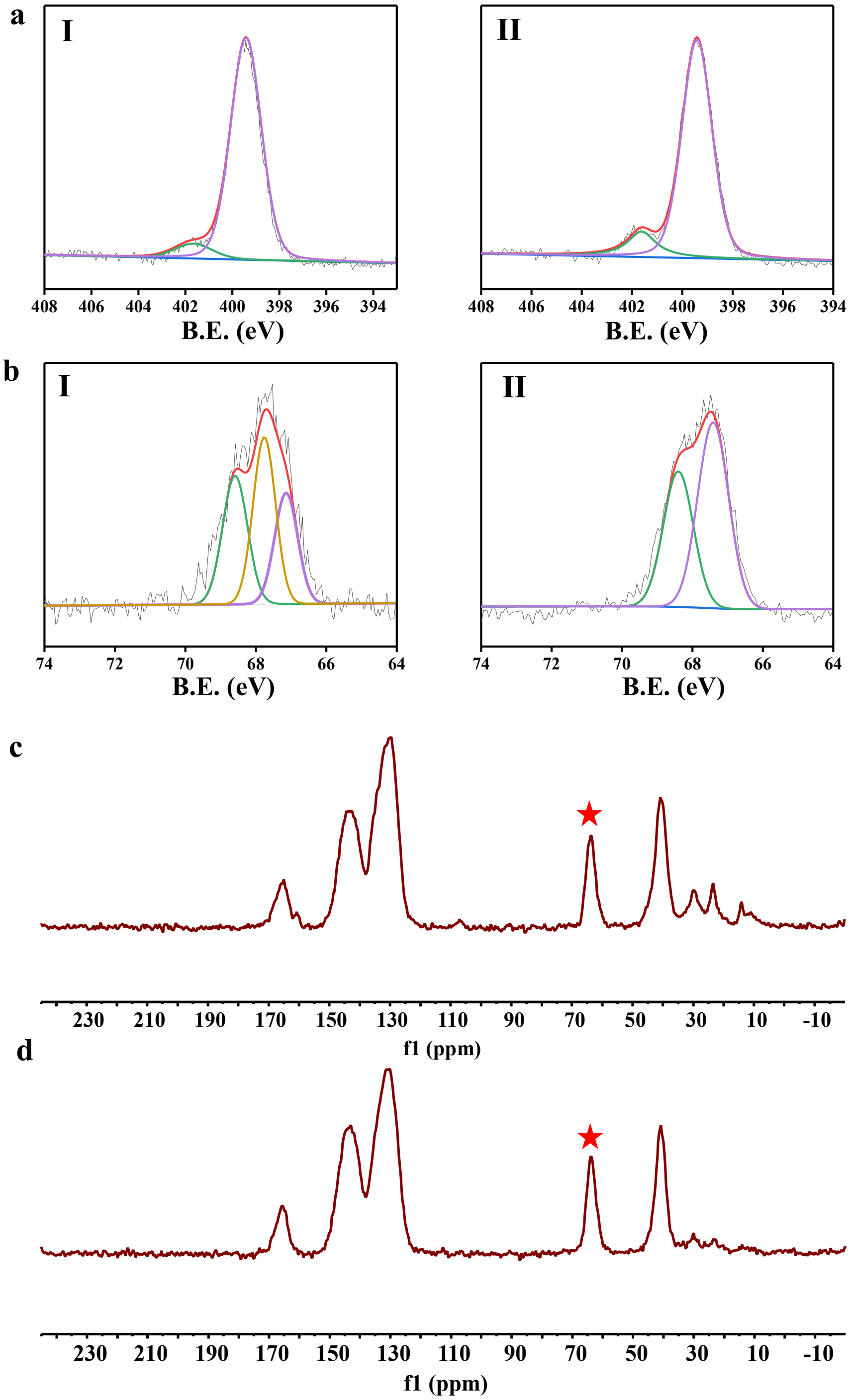

Supplementary Figure 12. (a) X-ray photoelectron spectra (*XPS*) of N (*1s* orbit) of **HOF1** (I) and **HOF2** (II). (b) *XPS* of Br (*3d* orbit) of **HOF1** (I) and **HOF2** (II). The corresponding degree of polymerization was further confirmed as 1/5.0, 1/3.4 by *XPS* measurements, which was consistent with the theoretical value indicating that the pyridines on the porous surface reacted with BBMB. Solid-state ^{13}C NMR of **HOF1** (c) and **HOF2** (d) (Red star in two frameworks is the C-Py^{+} resonance after cross linking) **(HOF**: hydrogen-bonded porous framework).

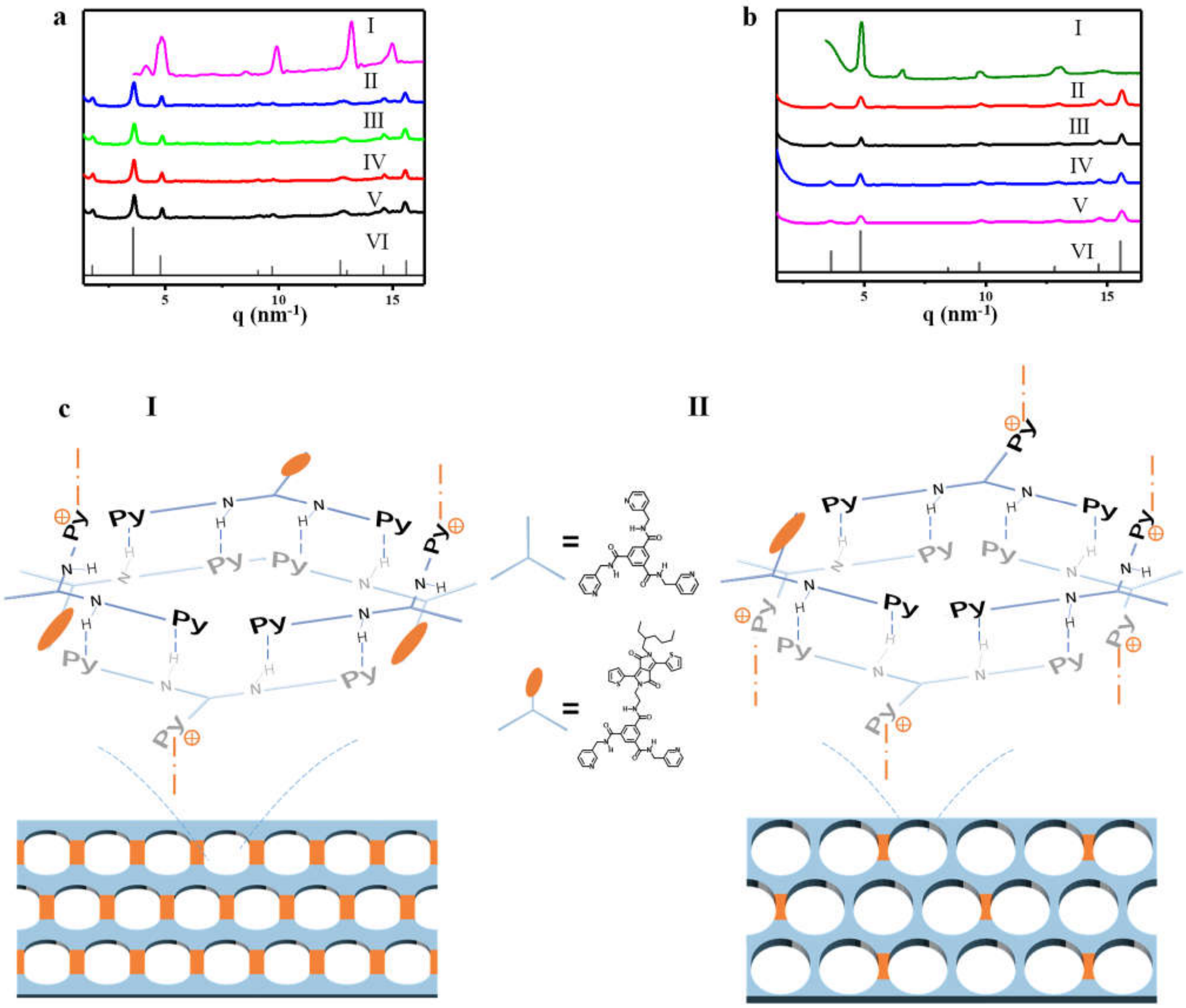

Supplementary Figure 13. X-ray diffraction of **HOF1** (a) and **HOF2** (b) (Initial sample before (I) and after (II) cross linking, III: after 4 h light irradiation, IV: soaked in HCl [pH 3] solution, V: soaked in NaOH [pH 11] solution). VI: the results of structure simulation according to Bragg's equation. (c) Schematic representation of **HOF1** (I) and **HOF2** (II) by the cross-linking of RPL and HPL structures **(HOF**: hydrogen-bonded porous framework, **RPL**: rectangularly perforated layers, **HPL**: hexagonally perforated layers).

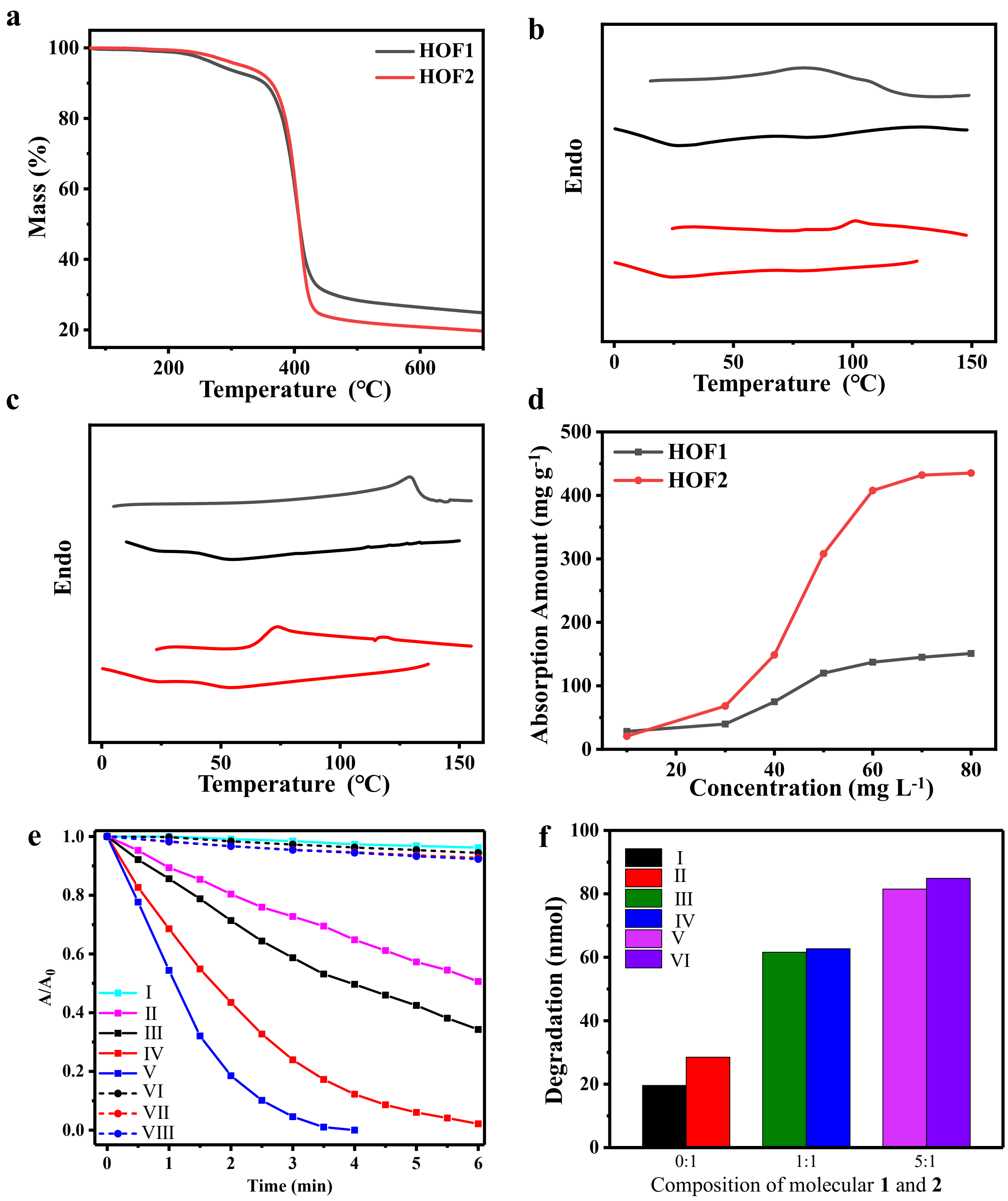

Supplementary Figure 14. (a) TGA curves of **HOF1** and **HOF2**. DSC curves recorded during the first (black) and second (red) heating and cooling scan of **HOF1** (b) and **HOF2** (c). (d) Adsorption isotherm of **HOF1** and **HOF2** for benzaldehyde. (e) Degradation rates of ABDA by dissolved **1** (I), **2** (II); self-assembled **2** (III), **RPL** (IV) and **HPL** (V); the aggregated **2** (VI), **RPL** (VII) and **HPL** (VIII) with NaN_3. Conditions: all structures contained same TDPP segments with the concentration of 0.1 μmol except **1**. (f) relative efficiencies of $^1O_2$ generation of **2** in dissolved solution (I) and self-assembled solution (II), **HOF1** before (III) and after (IV) cross-linking, **HOF2** before (V) and after (VI) cross-linking upon light irradiation for 2 min (**HOF**: hydrogen-bonded porous framework, **RPL**: rectangularly perforated layers, **HPL**: hexagonally perforated layers).

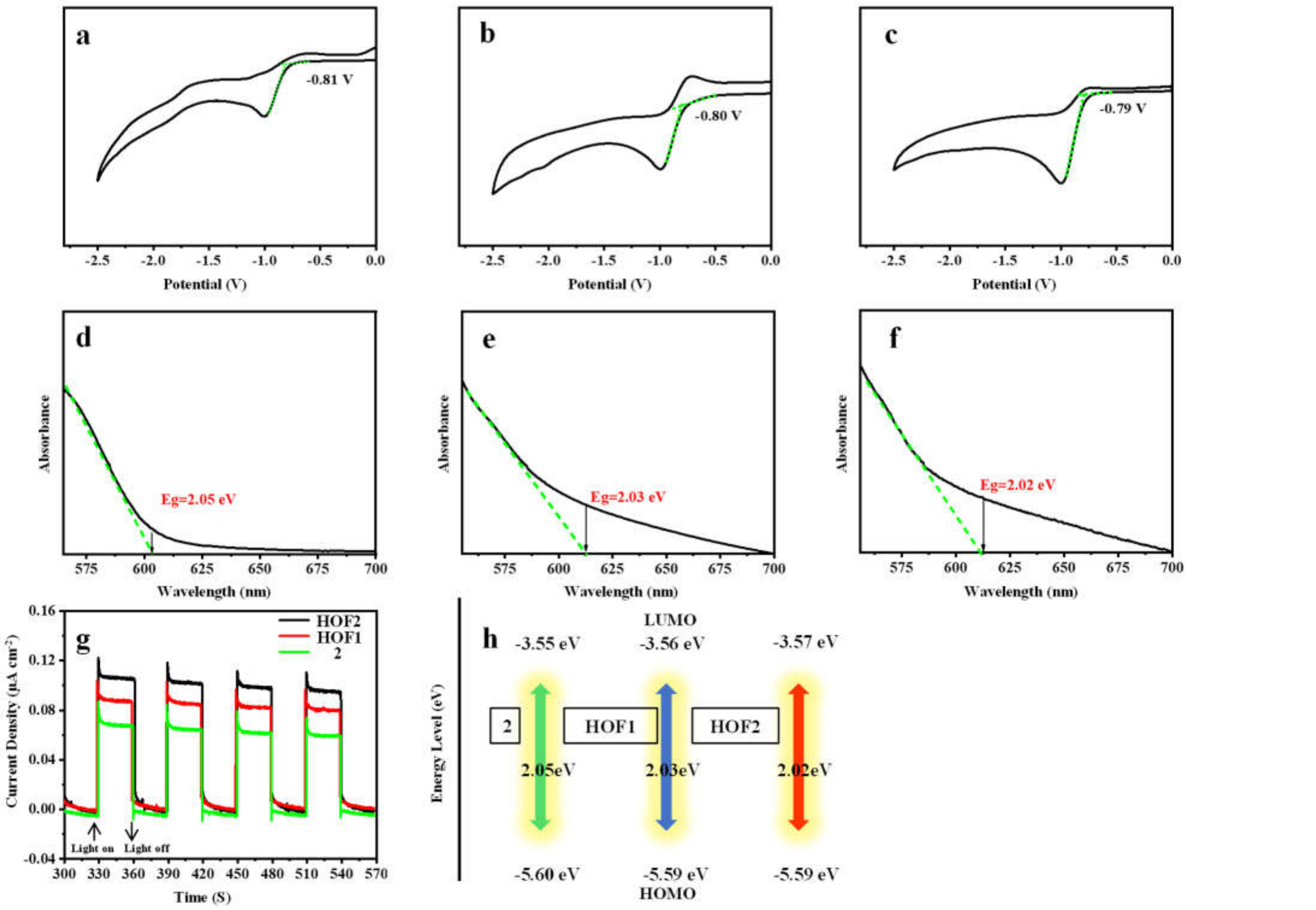

Supplementary Figure 15. Cyclic voltammograms of **2** (a), **HOF1** (b) and **HOF2** (c) in 0.1 M Bu_4NPF_6 acetonitrile solution. UV-vis spectra of **2** (d), **HOF1** (e) and **HOF2** (f). (g) Transient photocurrent response of **2**, **HOF1** and **HOF2** under intermittent irradiation. (h) The relative energy level of **2**, **HOF1** and **HOF2** calculated from the curve of cyclic voltammogram and observed band gap **(HOF**: hydrogen-bonded porous framework).

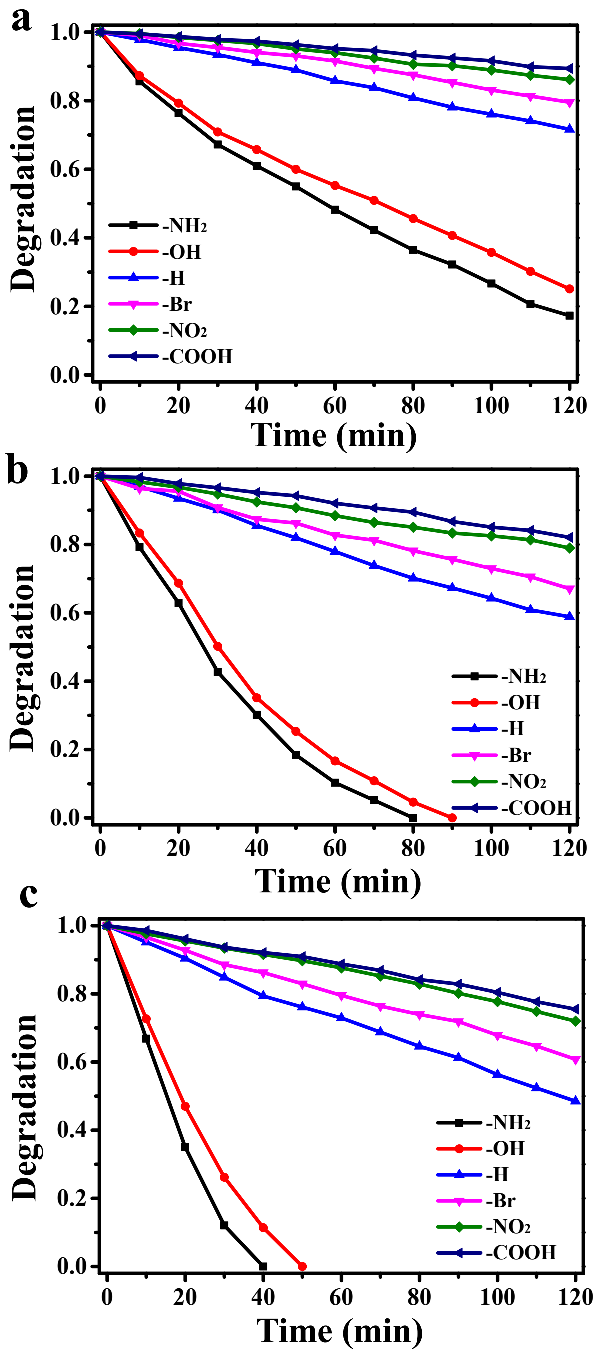

Supplementary Figure 16. The degradation efficiency of pyrene and its derivatives with catalyst **2** (a), **HOF1** (b), **HOF2** (c). For the comparison all catalysts contained 0.1 μmol PS segments **(HOF**: hydrogen-bonded porous framework).

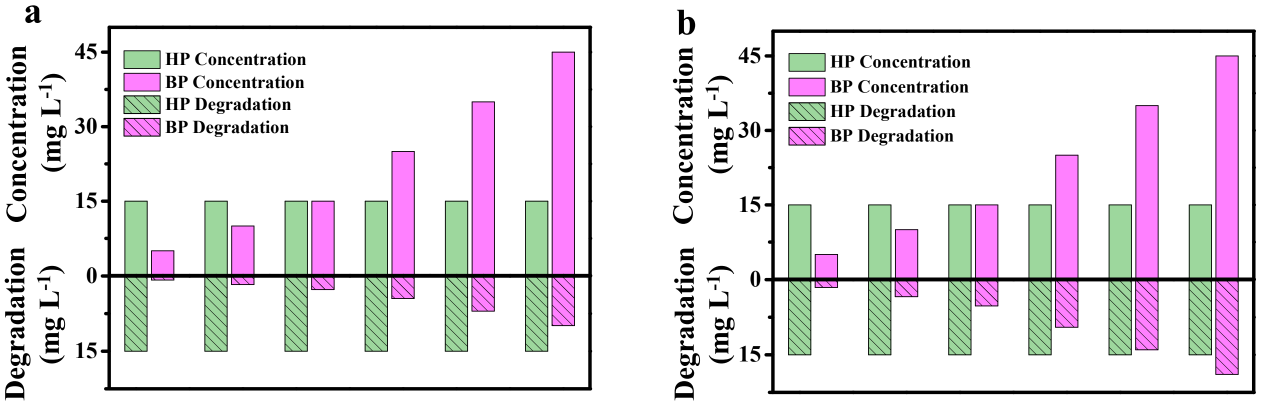

Supplementary Figure 17. The degrading comparison of HP and BP in the presence of **HOF1** (a) and **2** (b) by the increasing BP quantity **(HOF**: hydrogen-bonded porous framework).

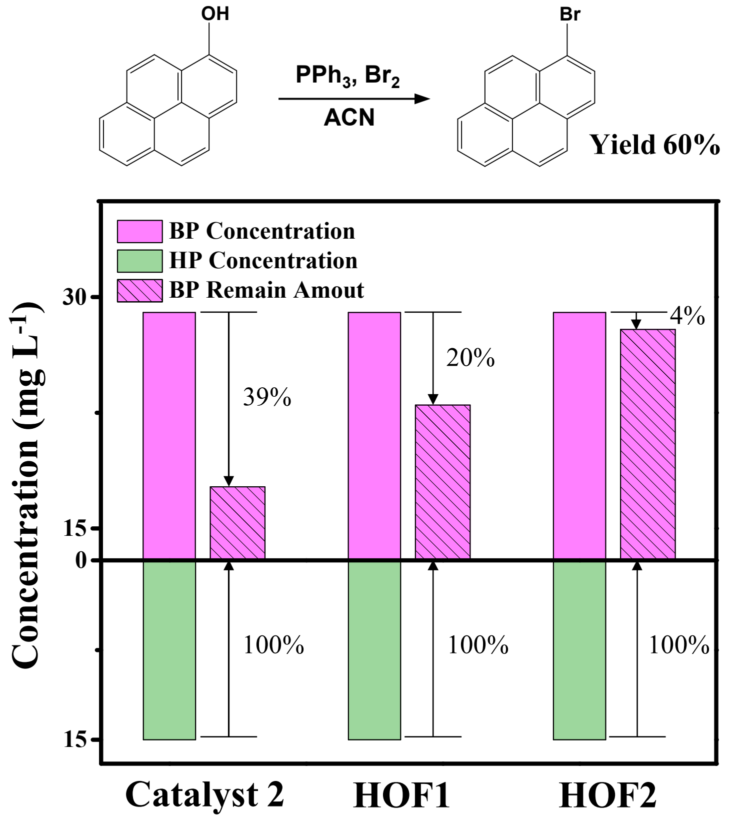

Supplementary Figure 18. The photocatalytic degradation of resulted bromo-substitution in the presence of catalyst **2**, **HOF1** and **HOF2** up to the decomposition of reagent **(HOF**: hydrogen-bonded porous framework).

Supplementary Tables

Supplementary Table 1. Elemental analysis results of **HOF1** and **HOF2**.

		**C%[a]**	**H%[a]**	**N%[a]**	**S%[a]**	**N/Br ratio[b]**
**HOF 1**	Calcd.	63.37	5.11	12.64	4.45	13/1
	Found	61.80	5.24	12.29	4.31	13/1
**HOF 2**	Calcd	61.74	4.79	13.32	1.65	7.4/1
	Found	59.39	5.04	12.71	1.56	7.1/1

[a] Data based on C, H, N, S elemental analysis.

[b] Data obtained from *XPS* analysis.

References

1. Li, Y. G. et al. Precisely controlled multidimensional covalent frameworks from the polymerization of supramolecular colloids. Angew. Chem. Int. Ed. 59, 21525-21529 (2020).
